# Recent progress in electrochemical assessment of DNA based on nanostructured sensors

**DOI:** 10.1007/s10544-025-00763-0

**Published:** 2025-07-12

**Authors:** Lue Wang, Waye Zhang

**Affiliations:** 1 Department of Chemical Engineering, Swansea University, Swansea, SA1 8EN UK; 2 School of Pharmaceutical Sciences, Tsinghua University, Beijing, 100084 China

**Keywords:** Electrochemical, DNA, Nanosensors

## Abstract

Screening the amount of DNA closely related to early diagnosis of diseases or decoding information in target DNA sequences for biological medicine, infectious identification, or forensic analysis are highly essential in our daily life. This review provides clear understanding of nanostructured sensors (i.e., functionalized electrode-based sensors and nanopores) working for electrochemical assessment of DNA, along with their recent advances and unaddressed issues. Crucial constituents for sensor functionalization, electrochemical techniques, and electrodes, used in functionalized electrode-based sensors are briefly introduced, followed by analysis of using this type of sensors for DNA determination and the comparison of performances such as dynamic ranges and detection limits with other similar works. Subsequently, nanopore sensors including porin-based and solid-state nanopores applied for DNA sequencing are the other interests of discussion in the review. Beyond the achievement of high-resolution DNA sequencing based on porins coupled with enzymatic components, commonly used methods to solid-state nanopore creation, practical use of solid-state nanopores in DNA analysis, and computational modeling for nucleobase pore-threading simulation are depicted in more detail. Finally, conclusions in relation to recent advances and future developments are described. This work offers a powerful guideline for electrochemical assessment of DNA using either functionalized electrode-based sensors or nanopores, enabling scientific groups to have an entire picture upon electrochemical nanodevices used for DNA characterization.

## Introduction

Deoxyribonucleic acid (DNA) molecules are basic constituent units in most organisms. DNA sequences contain different but specific biological information, which are used to guide various living activities. Electrochemical assessment of DNA molecules has a close association with genetic engineering, genomic studies, and structural prediction of proteins or proteinaceous molecules, as well as medical diagnoses and foodstuff quality monitoring (Castro-Wallace et al. 2017). To make determination more facile, accurate, and sensitive, various sensing techniques have been developed. The detection of DNA through electrochemical nanostructured sensors is more promising by virtue of rapid responses, simple manipulation, device portability, cost effectiveness, and mass production (Ronkainen et al. 2010).

**Fig. 1:**
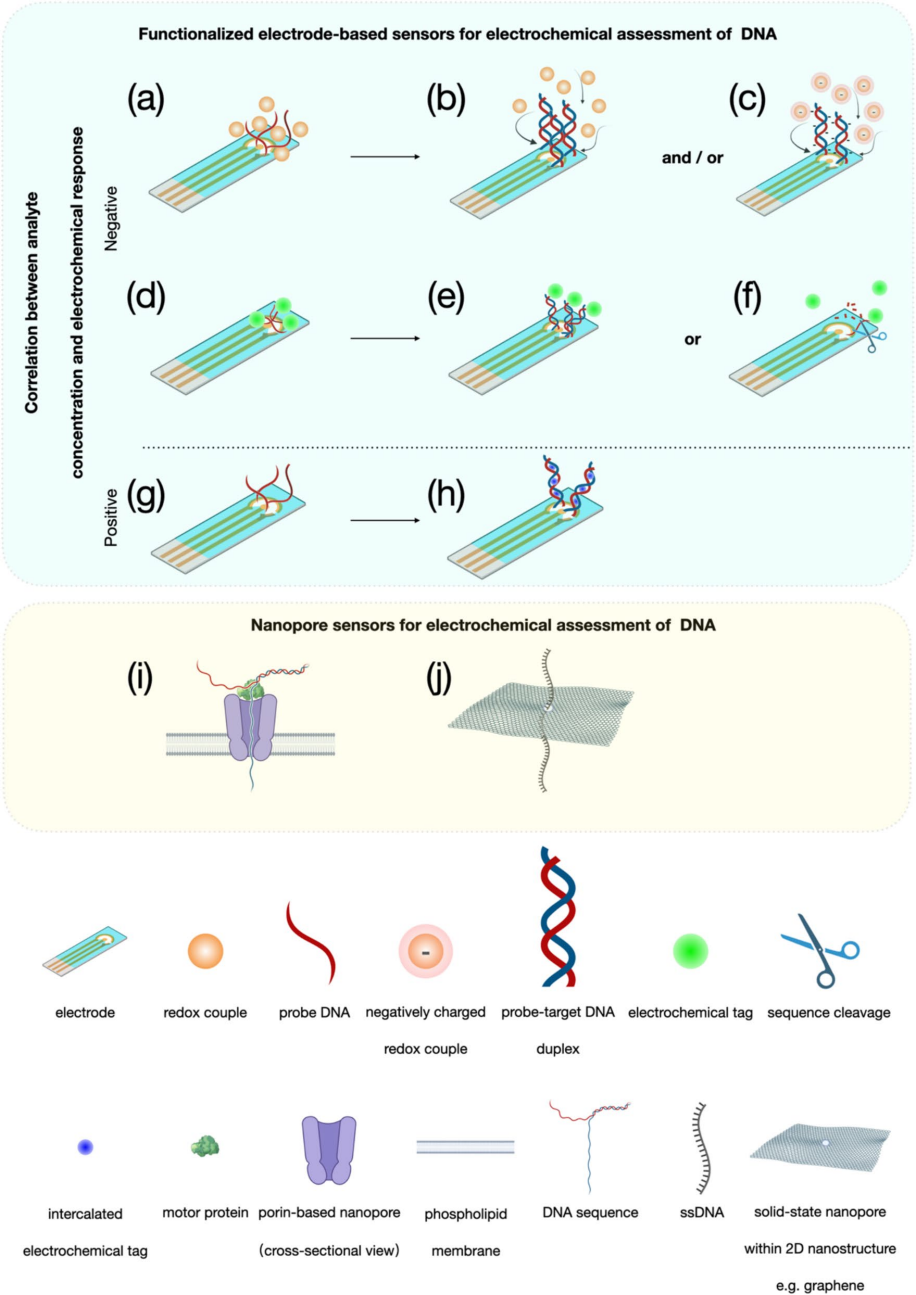
Functionalized electrode-based sensors for electrochemical assessment of DNA: **a** electrode surface with DNA probes only, redox couples move towards against less resistance; **b** electrode surface with probe-target DNA duplex, increased steric hinderance causes negative correlation between DNA analyte and electrochemical response; **c** negative correlation caused by electrostatic repulsion between negatively charged redox couple and DNA sequence; **d** electrochemical tags adhere to electrode surface modified with DNA probes only; **e** negative correlation caused by increased distance between electrochemical tags and electrode surface after probe-target DNA duplex formed; **f** negative correlation caused by electrochemical tags away from electrode surface owing to sequence cleavage; **g** electrode surface with DNA probes only, providing no room for electrochemical tag intercalation; **h** electrode surface with probe-target DNA duplex, electrochemical tags intercalated into structural gaps leading to positive correlation between DNA analyte and electrochemical response; **i** Porin-based sequencing, DNA sequence threads through nanopore at lower speed controlled by motor protein; **j** ssDNA passes through solid-state nanopore

Electrochemical nanodevices applied for DNA monitoring usually contain functionalized electrode-based sensors and nanopores. For functionalized electrode-based sensors, outstanding characteristics of high conductivity and adequate bioactive sites make the sensing interface appropriate to anchor enough ‘baits’ of target molecules (e.g., probe DNA strands), thereby reaching a sensitive detection. After forming double-helix structures with target complements, various impacts upon the electron pathway are immediately triggered, such as electron transfer promotion due to intercalation of electrochemical tags (Fig. 1g, h), or the other way around, charge transfer resistance (Fig. 1a, b) or electrostatic repulsion (Fig. 1a, c) against redox couples, or enabling electrochemical tags away from the electrode interface (Fig. 1d, e, f), thereby causing electrical signals linearly vary with concentrations of target analytes. As for measuring ensembles, the usage of three-electrode system remains its dominance in concentration determination of target molecules after the integration with functionalized electrode-based sensors. Normally, it contains a working electrode (e.g., DNA probe covered chips), a counter electrode, sometimes also called auxiliary electrode (e.g., platinum) and a reference electrode (e.g., Ag/AgCl). Latest advances about functionalized electrode-based sensors for the electrochemical DNA assessment reveal that two-dimensional (2D) nanomaterials such as graphene oxide nanoribbons (GONR) (Pareek et al. 2023), transition metal carbides (e.g., MXene Ti_3_C_2_) (Duan et al. 2023) are often introduced onto the electrode surface, aiming to offer tremendous room for DNA probe immobilization because of the increased surface-area-to-volume ratio. Furthermore, electrochemical sensors coupled with enzymatic components such as clustered regularly interspaced short palindromic repeats associated proteins (CRISPR-Cas 12a) (Duan et al. 2023; You et al. 2023), restriction endonuclease EcoRI (Meftah et al. 2023) have been receiving more interest owing to the remarkable enhancement of sensitivity. Although functionalized electrode-based sensors for electrochemical DNA determination are mostly used in fields of cancer biomarker monitoring, environmental protection, and food security, the monotonous function of concentration quantification hinders this type of sensor further development.

It is noteworthy that studies on the use of nanopores for assessment of biological molecules also belong to the field of electrochemical detection, however studies on porin-based nanopores themselves are often defined as electrophysiology. The review pays more attention to actual application of nanopores rather than focusing on nanopores themselves. Simple nanochannels coupled with electrophoretic forces always make nanopores exhibiting stronger versatility over other nanodevices. Many substances in fact can be electrochemically detected using nanopores such as DNA (Laszlo et al. 2014), ribonucleic acid (RNA) (Soneson et al. 2019), small molecules (Nestorovich et al. 2002; Fan et al. 2024; Sun et al. 2025), and peptides (i.e., precursors of proteins) (Wanunu 2022). Sensing of DNA molecules based on nanopores, in most cases, is described as a more pertinent term as “DNA sequencing” since nucleobase-dependent ionic current drops are created when a sequence of DNA with random base ordering threads through the constriction domain of a nanopore subjected to an electric field force. Many scientific or clinical studies have been successfully conducted since the company of Oxford Nanopore Technologies (ONT) first turned laboratorial DNA nanopore sequencing techniques into commercial applications. Versatile detecting of microRNA, biomarkers, and small proteinaceous substances via the reading of DNA-barcoded probes is recently reported based on the MinION sequencing device, one of nanopore products from the ONT, which significantly expands the practical use of nanopore technique (Koch et al. 2023). To reach clear-cut ionic current signals, Mereuta et al., reported a novel method to ssDNA sequencing based on the α-hemolysin (α-HL) nanopore, where negatively charged AuNPs coupled to charge neutral peptide nucleic acids (PNA) were applied to specifically recognize target ssDNA strands (Mereuta et al. 2020). Another approach to improving the signal fidelity of DNA is to develop porin-based nanopores with dual constriction (Verren et al. 2020). However, only DNA homopolymer sequences were assessed through the system. Conventional DNA sequencing based on porins always introduces motor proteins such as DNA polymerase or helicase to enable the DNA strand to move at the pace of individual base when threading through the nanopore. However, enzymatic components are limited by various ambient factors such as temperature, solution pH, or osmotic pressure, which are not very suitable for all environments. Facing this challenge, Qing and Bayley reported a method to DNA identification via chemical stepping instead of enzyme-based sequencing (Qing and Bayley 2021). Since the end of 2019, the COVID-19 pandemics have caused serious health threat to the people all over the world, making the technique of DNA sequencing highly useful for virus infection confirmation (Stüder et al. 2021). Beyond this application, nanopore-based DNA sequencing can be also applied in analysis of single-molecule methylation of cell-free DNA for early warning of cancer, or various classes of DNA methylation from microbiomes and bacteria (Lau et al. 2023; Tourancheau et al. 2021). Although, up to date, high resolution DNA sequencing has only been realized on porin-based nanopores (Fig. 1i), there are still a couple of issues often occurring during the formation of lipid membrane. Like enzymes, the form of double-layered lipid membrane also requires rigorous conditions such as appropriate temperature and humidity, while unproper parameters employed prone to result in failure of membrane formation. Additionally, porin-based nanopores are almost all transmembrane proteins, which periodically require complicated protein expression and purification for one-batch mass production, leaving lengthy pre-preparation prior to the formal sequencing assay. Solid-state nanopores, someday, may replace porin-based nanopores owing to unique advantages of low cost, simple fabrication, excellent reusability, good stability, and free design on nanopore sizes (Fig. 1j) (Xue et al. 2020). The sophisticated porin preparation, lipid membrane coverage on a micro-aperture-containing support, and porin embedment are no longer required when using solid-state nanopores, thus avoiding the risk of membrane broken at any unexpected moment. Notwithstanding, the poor signal-to-noise ratio (SNR), lack of active sites for assigned anchoring of biological components nearby a solid-state nanopore, and difficulty in fabrication of solid-state nanopores with atomic-grade consistency, all make this type of nanopore not feasible yet for high-resolution DNA sequencing. Scientific studies in relation to DNA sequencing based on solid-state nanopores currently concentrate on machine learning or computer-assisted modeling to understand or provide interpretation on DNA translocation through a solid-state nanopore (Jena et al. 2022). Breakthroughs regarding practical use of solid-state nanopores are not too much. The nanopore developed by pulling a quartz capillary has received an increasing amount of attention in recent years due to cost effectiveness, simple fabrication, and potential for large-scale production. However, sectional diameters of these artificially conical nanopores are about 3 ~ 5 nm, or even more, which are double- or multiple- fold wider than general ssDNA strands (Chen et al. 2021, 2023). Choudhary et al., created a nanofluidic system consisting of dual nanopores for single-molecular DNA sequencing, aiming to study behaviors of DNA capturing and threading with high fidelity (Choudhary et al. 2020). While for high-resolution reading of DNA, the setup requires further development in the pore size reduction from current 10 nm to 1 nm.

Interestingly, the discrimination of target DNA from other interferents can be realized via both classes of nanostructured sensors. Functionalized electrode-based sensors allow target DNA molecules to specifically bind to an electrode surface, yielding distinctively electrochemical responses that are always several orders of magnitude higher or lower than those of other interferents. While nanopores monitor base-dependent ionic current blockages as DNA sequences transverse through a nanopore, achieving target DNA recognition by virtue of the unique readout different from other interfering sequences. This review mainly focuses on two types of DNA nanostructured sensors (i.e., functionalized electrode-based sensors or nanopores) in combination with electrochemical techniques. Miscellaneous nanodevices prepared, along with their sensing performances such as dynamic ranges and detection limits are carefully analyzed, with further development or prospects for two types of DNA nanostructured sensors provided at the end of the review. This work aims to offer study groups or companies a powerful scientific support in electrochemical sensing based on functionalized electrode-based sensors or nanopores.

### Electrochemical assessment of DNA using functionalized electrode-based sensors

Functionalized electrode-based sensors are commonly used in quantitative analyses of target DNA analytes either in a buffered saline or a real biological solution (Table 1).

**Table 1:** Functionalized electrode-based sensors used for electrochemical assessment of DNA

DNA analytes	Electrode modification	Electrochemical techniques	Dynamic ranges and LODs (pM)	Purposes for electrochemical assessment	Refs
ARGs	GCE/4-ABA/EDC/NHS/probe DNA	EIS	21.6 ~ 477.12 and 21.6	Pollutants monitoring	Wan et al. 2023)
cDNA	LIG/C15 pDNA/23 random DNA	SWV	0.1 ~ 10^5^ and 0.057	DNA hybridization quantification	Bahri et al. 2023)
Target DNA	AuNWE/MCH/HS-DNA	SWV	10^–3^ ~ 10^4^ and 4.8 × 10^–4^	Target DNA quantification	Hua et al. 2018)
Target DNA	Graphene microfiber/ZnO NWs/probe DNA	EIS	0.01 ~ 10^5^ and 3.3 × 10^–3^	DNA hybridization quantification	Zhang et al. 2019)
Target DNA	GCE/GO/PANIw/probe DNA	DPV	2.12 ~ 2.12 × 10^6^ and 0.325	DNA-based diagnosis	Bo et al. 2011)
Genomic DNA	AuE/PEDOT-PSS/AuNPs/DPA/DNA reporter	DPV	3,130 ~ 10^5^ and 0.054	Detection of white rot fungus	Hushiarian et al. 2015)
RASSF1A tumor suppressor gene	SPCE/PT/anti-5mC antibody/methylated target strand	DPV	0.01 ~ 5,000 and 0.002	DNA methylation screening	Daneshpour et al. 2016)
HPV-16	SPCE/Fe_3_O_4_/AuNPs/thiolated probe DNA	DPV	0.1 ~ 10^6^ and 100	Early warning of cervical cancer	Rasouli et al. 2023)
HPV-16	ITO/GONR/Ag@AuNPs/probe DNA	LSV and EIS	10^–4^ ~ 10^6^ and 10^–4^	Early warning of cervical cancer	Pareek et al. 2023)
HPV-18	SPCE/MXene Ti_3_C_2_/AuNPs/ssDNA	SWV	10 ~ 5 × 10^5^ and 1.95	Early warning of cervical cancer	Duan et al. 2023)
IL6 and TGFβ1	SPCE/AuNPs/thiolated ODN probes	DPV	IL6: 0.1 ~ 10^5^ and 0.048 TGFβ1: 0.05 ~ 100 and 0.017	Early warning of ovarian cancer	Meftah et al. 2023)
Lung cancer biomarker DNA	AnE/SnO_2_-QD-Au/ssDNA	EIS	10^–8^ ~ 10^6^ and 3.2 × 10^–8^	Early warning of lung cancer	Richard et al. 2023)
*p53* gene	MGCE/Fe_3_O_4_/α-Fe_2_O_3_@Au/HS-PNA	DPV	1 ~ 10^6^ and 0.85	Early warning of human cancer	Liu et al. 2023)
*p53* gene	Au/MUC/ssDNA	CV	0.001 ~ 100 and 1.75 × 10^–4^	Early warning of human cancer	Garcia-Melo et al. 2023)
*S. flexneri* DNA	ITO/P-Mel/PGA/DSS/CP	DPV	10^–9^ ~ 10^6^ and 7.4 × 10^–10^	Food quality evaluation	Ali et al. 2023)
*stx1* and *stx2*	Si-Au IDEs/AuNPs/Cht-Au/probe DNA	SWV	*stx1*: 10^–7^ ~ 10^–2^ and 10^–7^ *stx2*: 10^–7^ ~ 10^–1^ and 10^–7^	Food quality evaluation	Wasiewska et al. 2023)
Base G and A	N/P/S doped graphene paper/dsDNA	CV	G: 4 × 10^5^ ~ 4 × 10^6^ and 1.5 × 10^6^ A: 4.4 × 10^5^ ~ 4.4 × 10^6^ and 2.38 × 10^6^	Exceptional biomarker detection	Jagannathan et al. 2023)
Base G and A	SPCE/f-CB/glass microbead with probe DNA	DPV	G: 5 × 10^5^ ~ 10^7^ and 2.8 × 10^5^ A: 5 × 10^5^ ~ 2 × 10^7^ and 1.78 × 10^6^	Exceptional biomarker detection	Aamri et al. 2023)
r60	Au/MCH/MUA/NHS/DANP/probe DNA	EIS	10^4^ ~ 10^7^ and 10^4^	Target DNA quantification	Han et al. 2023)
KRAS G12D	SPE/probe DNA	CV	10^–6^ ~ 1 and 10^–5^	Target DNA quantification	You et al. 2023)
*M. Smegmatis* DNA target	C223AT-SPE/MCP/16S rRNA probe	EIS and SWV	-	Detection of antibiotic susceptibility	Güzel et al. 2021)

### Functional or bio-functional modification on sensor surfaces

The functional or bio-functional modification with nanostructures or nanocomposites on electrode surfaces is considered as an indispensable fabrication process since it helps in enlarging the surface area for accommodation of bioactive sites, leading to improved sensitivity and specificity in electrochemical sensing. Functional monomers such as 4-aminobenzoic acid (4-ABA) (Wan et al. 2023), melamine (Mel), and glutamic acid (GA) (Ali et al. 2023), are often introduced on the surface of electrode, creating a biocompatible coverage via an electro-polymerization for immobilization of DNA probes. As functional layers, self-assembled monolayers (SAMs) play a vital role in fabrication of electrochemical sensors as their multiple properties including hybridization promotion, non-specific site blocking, parallel orientation of DNA strands, and electrode lifetime improvement (Rasanang et al. 2022; Bahner et al. 2018; Ting et al. 2009; Nguyen and Minteer 2015; Lee et al. 2014). Commonly used SAMs include 11-mercaptoundecanoic acid (MUA) (Garcia-Melo et al. 2023; Han et al. 2023) and 6 carbon mercaptan (MCH) (Han et al. 2023). Carbon nanomaterials are another class of important functional layers for electrodes. Graphene oxide nanoribbons (GONR) (Pareek et al. 2023) are used for increasing the surface-area-to-volume ratio are often covered on the electrode surface through an electrodeposition method. Functionalized carbon black (f-CB) is a class of CB that contains organic groups (e.g., carboxylic group (Aamri et al. 2023)), which is usually prepared into a dispersed solution drop-casted on surface of electrodes serving as a highly conductive underlayer friendly to biomolecule binding. Similarly, oxidized graphene dispersion is also required to undergo a drop-casting process to form a uniform layer on glassy carbon electrodes (GCE), offering a good environment for immobilization of biomolecules or other nanostructures (Bo et al. 2011).

In some cases, DNA probes are not able to directly attach to the functionalized electrode surface unless there is a cross-linking agent involved such as 1-ethyl-3-(3-dimethyl aminopropyl) carbodiimide (EDC) coupled to N-hydroxy succinimide (NHS) (Wan et al. 2023), disuccinimidyl suberate (DSS) (Ali et al. 2023), and 3,3’-dithiopropionic acid (DPA) (Hushiarian et al. 2015). Multiplex linkers are also used in some studies allowing electrode surfaces to have specific affinity for DNA probes such as NHS coupled with 2, 7-diamino-1, 8-naphthyridine (DANP) (Han et al. 2023) that prefers adsorption of cytosine bulge structures. Moreover, poly-cytosine (poly-C) DNA strands (e.g., C15 pDNA) are sometimes applied as a special bio-linker owing to the strong adsorption to carbon nanostructures (e.g., graphene) or other inorganic nanomaterials (Bahri et al. 2023). Nanoparticles, another class of frequently utilized nanostructures, are regarded as one of key components in surface biofunctionalization of electrochemical nanosensors. Gold nanoparticles (AuNPs) are mostly used nanoparticles capable of introducing various bonds such as the gold-thiol (Hua et al. 2018) or gold-streptavidin (Daneshpour et al. 2016) conjugation for covalently bonding with DNA probes. Interestingly, AuNPs in combination with other metal or metal oxide nanoparticles such as silver and ferroferric oxide nanoparticles (Fe_3_O_4_ NPs) can form nanoparticle-based composites as silver coated gold nanoparticles (Ag@AuNPs) (Pareek et al. 2023) and Fe_3_O_4_-Au core–shell nanoparticles (Rasouli et al. 2023), respectively. The increased surface area allows more DNA probes anchoring on the electrode functionalized with nanoparticle-based composites, compared with that of untreated electrode surfaces. Owning a strong magnetic feature, ferroferric oxide nanoparticles (Fe_3_O_4_ NPs) can be also integrated with DNA probes such as magnetic nanoparticle-capture probes (MNP-Capture probes) (Hushiarian et al. 2015), which can be used for target DNA enrichment. Quantum dots (QD) such as SnO_2_-QD (Richard et al. 2023), belong to a special class of nanoparticles with geometrical size less than 100 nm. They have been recently applied in the process of electrode fabrication to achieve a downsized device with higher sensitivity and selectivity (Kolhe et al. 2023; Hansen et al. 2006). Nanowire-based structures play essential roles in electrochemical characterization of DNA molecules. Sensing devices with polyaniline nanowires (PANIws) are of particular interest, since polyaniline possesses strengths including straightforward fabrication, large surface area, and tunable conductivity. (Maynor et al. 2002; Wu and Bein 1994; Noll et al. 1998) Zinc oxide nanowires (ZnO NWs), a nanomaterial with a broad band gap of 3.37 eV, recently has been applied in sensor fabrication (Cao et al. 2016; Kong et al. 2009; Tereshchenko et al. 2016). The growth of ZnO NWs on various substrates can be easily achieved via a hydrothermal method (Zhang et al. 2019). Additionally, the positively charged surface is of non-negligible characteristic upon ZnO NWs, which directly attaches negatively charged probe DNA strands to the surface through electrostatic interaction (Zhang et al. 2019).

### Electrochemical techniques and electrodes

Electrochemical techniques suitable for functionalized electrode-based sensors consist of voltammetric, amperometric and impedimetric categories. Voltammetric measurements provide information of current variation over an assigned range of potential, where peak currents normally represent oxidation or reduction level of signal indicators. Amperometric measurements offer a picture of current variation versus time, and information of tested concentrations can be directly indicated according to a series of current levels. Impedimetric measurements can convert the actual microenvironment of electrode-solution interface into an equivalent circuit, of which impedance signals vividly reflect the biochemical events as they usually comprise of a real part (i.e., charge transfer resistance) and an imaginary part (i.e., capacitance). Electrochemical techniques for assessment of DNA on functionalized electrode-based sensors are mostly voltammetric measurements such as cyclic voltammetry (CV) (You et al. 2023; Garcia-Melo et al. 2023; Jagannathan et al. 2023), differential pulse voltammetry (DPV) (Meftah et al. 2023; Bo et al. 2011; Hushiarian et al. 2015; Daneshpour et al. 2016; Rasouli et al. 2023; Liu et al. 2023; Ali et al. 2023; Aamri et al. 2023), square wave voltammetry (SWV) (Duan et al. 2023; Bahri et al. 2023; Hua et al. 2018; Wasiewska et al. 2023), and linear sweep voltammetry (LSV) (Pareek et al. 2023). As the typical technique of impedimetric measurements, electrochemical impedance spectroscopy (EIS) is another effective approach to characterizing DNA at trace amount (Pareek et al. 2023; Wan et al. 2023; Zhang et al. 2019; Richard et al. 2023; Han et al. 2023). The use of EIS recent years has received a growing number of attentions owing to powerful properties of simple program setting, rapid analysis, high sensitivity, and setup miniaturization. Amperometric measurements in contrast are less reported in the determination of DNA. This is probably due to the mechanism not suitable for low concentration monitoring.

**Fig. 2:**
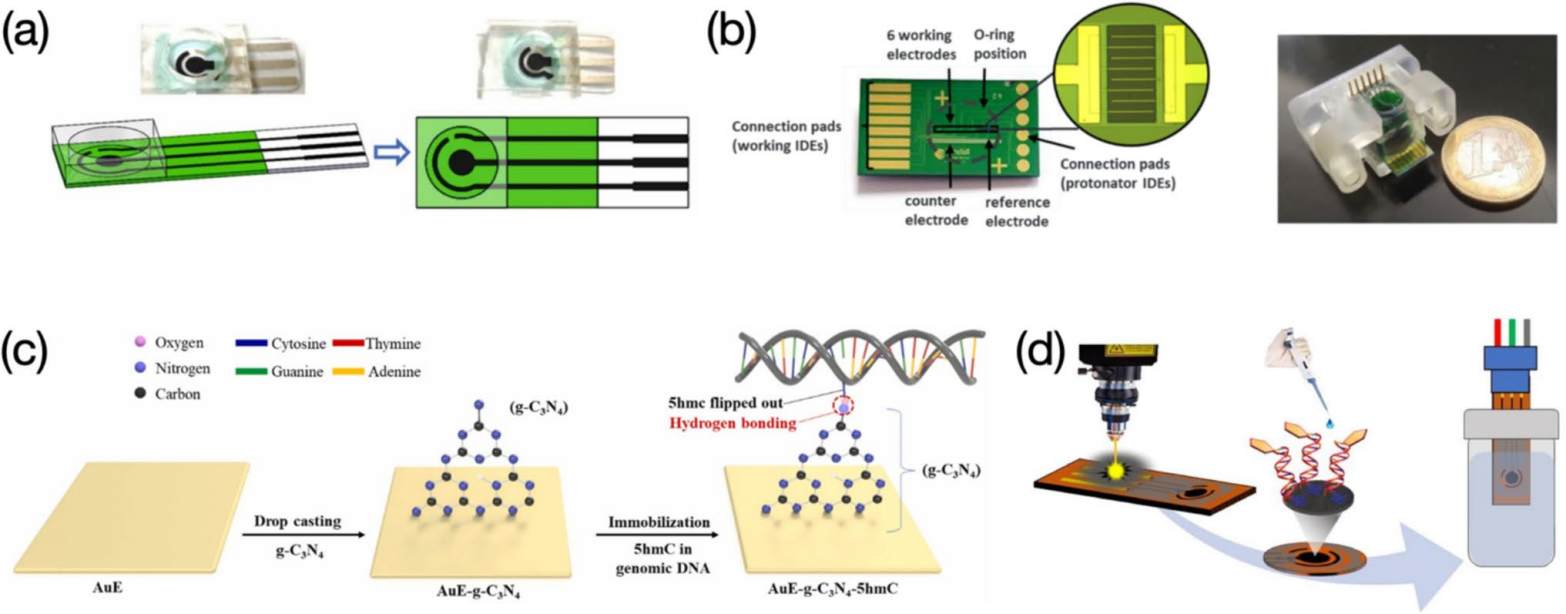
Various electrodes used for electrochemical assessment of DNA: **a** SPCE electrode coupled with a PDMS cover forming a confined sensing window. Image reprinted with permission from Yuan et al. (2023); **b** structure of Si-Au IDE along with the chip inserted in a holder. Image reprinted with permission from Wasiewska et al. (2023); **c** AuE attached with g-C3N4. Image reprinted with permission from Imran et al. (2023); **d** LIG electrode integrated with a three-electrode electrochemical sensing system. Image reprinted with permission from Bahri et al. (2023)

Electrodes are also the important component in electrochemical sensing systems as they play a role in electron transportation from bioactive interfaces to signal transformation devices. For functionalized electrode-based sensors, screen-printed carbon electrodes (SPCE) (Duan et al. 2023; Meftah et al. 2023; Daneshpour et al. 2016; Rasouli et al. 2023; Aamri et al. 2023) or gold electrodes (Hua et al. 2018; Hushiarian et al. 2015; Richard et al. 2023; Garcia-Melo et al. 2023; Han et al. 2023) are mostly used electrodes for electrochemical detection of DNA. SPCEs are usually designed as “three route” pattern catering for the three-electrode system, of which the sensing window is always with a concentric shape to maximumly increase the effective contacting area. Other than SPCEs, the screen-printing technique can be also utilized in fabrication of electrodes with gold, forming screen-printed gold electrodes (SPGE) (You et al. 2023). Polydimethylsiloxane (PDMS) films are sometimes used to cover around the sensing window of SPCEs to form a cylindrical chamber avoiding the testing solution to spread out of the electrode edge (Fig. 2a) (Yuan et al. 2023). In comparison to simple gold electrodes, interdigitated gold microelectrodes engraved at the surface of silicon chip (Si-Au IDEs) have sophisticated patterns capable of simultaneously detecting multiple DNA sequences (Fig. 2b) (Wasiewska et al. 2023). A gold electrode coupled with a novel 2D material of graphitic carbon nitride (g-C_3_N_4_) is recently reported for real-time determination of 5-hydroxymethylcytosine (5hmC) in genomic DNA molecules. The use of g-C_3_N_4_ makes the sensor having highly selective “antennas” capturing target DNA sequences via hydrogen bonding (Fig. 2c) (Imran et al. 2023). Graphene-based electrodes are another commonly used electrodes applied for electrochemical assessment of DNA. Laser-induced graphene (LIG) electrodes possess similar designs like SPCEs, which are fabricated through a photothermal pyrolysis sourced from laser pulses (Fig. 2d) (Bahri et al. 2023).

**Fig. 3:**
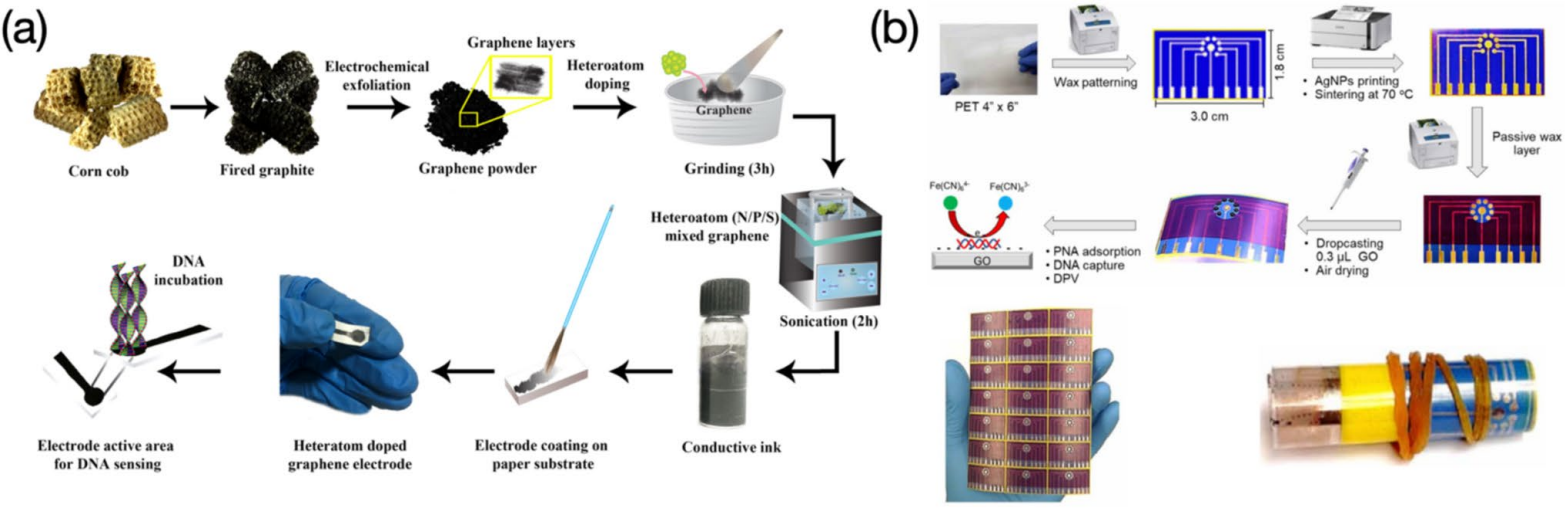
Flexible electrodes used for electrochemical analysis of DNA: **a** preparation workflow of graphene paper electrodes. Image reprinted with permission from Jagannathan et al. (2023); **b** fabrication process of wax-on-PET chips, along with its mass production and high flexibility. Image reprinted with permission from Das et al. (2023)

Flexible electrodes recently have earned a great amount of interest owing to their light weight, disposability, and low cost. Heteroatom doped graphene electrodes coated on a paper substrate have been used for rapid DNA detection, which the cost-effective preparation starts from corn cobs (Fig. 3a) (Jagannathan et al. 2023). Polyethylene terephthalate (PET) substrates subjected to wax patterning are made as an underlayer for deposition of AuNPs and graphene oxide (GO), yielding a multiplexed electrode for peptide nucleic acid (PNA) adsorption. The wax-on-PET chip presents good flexibility and can be massively produced from batch to batch (Fig. 3b) (Das et al. 2023). Other soft substrates such as indium tin oxide (ITO) (Ali et al. 2023) can also be made as flexible electrodes, however, ITO electrodes used for electrochemical analysis of DNA are less reported because of the high price of the material. Interestingly, there are also only a few reports on electrochemical determination of DNA using glassy carbon electrodes (GCE), while GCEs are top-preferred electrodes in other sensing applications (Wan et al. 2023; Bo et al. 2011; Liu et al. 2023).

### Negative correlation between electrochemical responses and target DNA concentrations

There are many studies in which resulting electrochemical outcomes present a negative correlation with concentrations of target DNA sequences. Such inversely proportional relationship can be explained as the electrostatic resistance or steric hinderance sourced from the probe-target DNA hybridization against the charge transfer of redox ion pairs.

**Fig. 4:**
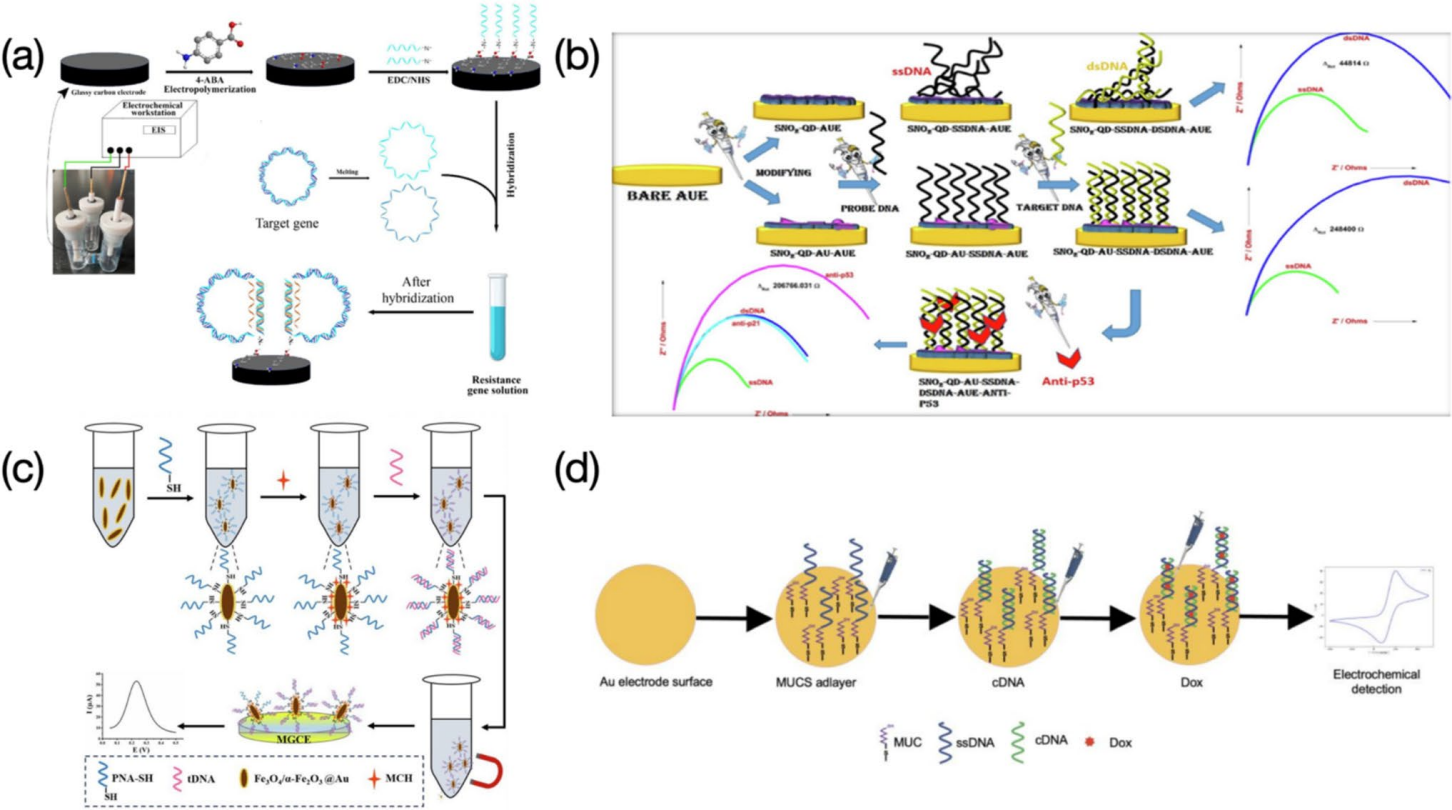
Schematic illustration of functionalized electrode-based sensors used for assessing DNA molecules, where electrochemical results are caused by steric hinderance sourced from double-stranded structures: **a** Impedimetric DNA sensor prepared for determination of ARGs. Image reprinted with permission from Wan et al. (2023); **b** EIS based electrochemical DNA sensor used for detection of lung cancer DNA biomarker and anti-p53 antibody. Image reprinted with permission from Richard et al. (2023); **c** A special “one-pot” sensing strategy used for detection of p53 genes. Image reprinted with permission from Liu et al. (2023); **d** Gold based electrochemical genosensor applied for detection of p53 gene mutation. Image reprinted with permission from Garcia-Melo et al. (2023)

Wan et al., prepared an electrochemical DNA sensor for sensitive determination of antibiotic-resistant bacteria (ARG) or resistance genes (ARGs) (Fig. 4a) (Wan et al. 2023). An increasing impedance against the electron transfer of [Fe(CN)_6_]^3−/4−^ was observed as more target genes were introduced and hybridized with DNA probes. The sensor presented a linear behavior towards ARGs with different concentrations ranging from 21.6 to 477.12 pM, reaching a detection limit of 21.6 pM. The strong environmental adaptability makes the sensor successful in detecting the target gene in actual sewage or river water specimens even interfering with ions and other pollutants. Richard et al. reported a versatile nanoelectrode used for label-free monitoring of lung cancer DNA biomarkers (Fig. 4b) (Richard et al. 2023). The use of AuNPs enables the electrode surface to have a uniform coverage of DNA probes, compared with the surface without AuNPs. Impedimetric responses produced by the sensor were observed in an increasing behavior as more target DNA molecules captured, reaching an extremely wide detection range of 10^–8^ ~ 10^6^ pM, coupled with an ultralow LOD of 7.4 × 10^–10^ pM. The probe-target DNA combination presented the highest charge transfer resistance over other groups including SnO_2_-QD-Au only, probe, non-complementary, and single base mismatch DNA, providing evidence of good selectivity. The sensor also exhibited outstanding electrode reproducibility in the charge transfer measurement for both single-stranded DNA (ssDNA) and double-stranded DNA (dsDNA) cases, along with a long-term stability of up to 21 days. Although target analytes were not always the biomarker for lung cancer in other studies in regard of cancer biomarker detection using electrochemical sensors, this work verified the technique of EIS sometimes can reach an ultrasensitive detection towards DNA strands. Beyond this, the sensor displayed an extra function of detecting anti-p53 antibody. Nevertheless, it worked only at the perfect dsDNA coverage. Liu et al. depicted a special “one-pot” sensing process for the detection of *p53* gene. The Fe_3_O_4_/α-Fe_2_O_3_ nanocomposite tethered with gold nanoparticles served as a substrate for immobilization of thiolated peptide nucleic acid (SH-PNA) probes and subsequent hybridization with target DNA, all in a single centrifuge tube (Fig. 4c) (Liu et al. 2023). 6-mercaptohexanol (MCH) was also involved and attached to the nanocomposite for blocking non-specific binding sites or pinholes. The resulting solution was finally cast onto the electrode surface to increase the steric hinderance against the redox couple, causing reduced DPV signals. The dynamic range regarding the *p53* gene detection based on the sensor was 1 ~ 10^6^ pM, with a LOD of 0.85 pM. The superior selectivity of the sensor made it capable of accurately differentiating single-, double-, and triple- base mismatched DNA sequences. The good reproducibility was demonstrated by testing seven electrodes with the target DNA, yielding current responses with a negligible relative standard deviation (RSD). Sensor stability was also studied, with the response at the twelfth day only presenting a 16.8% drop of the initial response. Furthermore, the target *p53* gene was spiked into human serum samples to assess the practicability of the sensor. The ultraviolent (UV) absorbance method was used to provide a double verification that the sensor is reliable in real clinical applications. Garcia-Melo et al. constructed a simple electrochemical genosensor for sensitive detection of the 175p2 mutation of the *p53* gene (Fig. 4d) (Garcia-Melo et al. 2023). In comparison to the detection without doxorubicin (Dox), the LOD was lowered by one order of magnitude in the presence of Dox, based on a detection range of 0.001 ~ 100 pM. Three non-complementary and the target DNA probes were tested using the sensor to evaluate the selectivity and only the target DNA probes presented reduced current signals after the hybridization for both cases of with or without Dox. Nonetheless, results on reproducibility, stability, and real sample testing were not discussed in the work. The proposed sensor was also compared to electrochemical nanodevices reported in other studies, confirming a relatively wide detection range and a lower LOD. The use of MB in the work of Meftah et al. (Meftah et al. 2023) results in greater currents measured as higher concentrations of target DNA are introduced, while decreased current reponses were observed when the other signal indicator of Dox is applied in this work. A relatively reasonable explanation is the introduction of Dox causes the compaction of the superficial layer which alters the spatial structure of double-helix complexes near the electrode surface (Malanina et al. 2022).

**Fig. 5:**
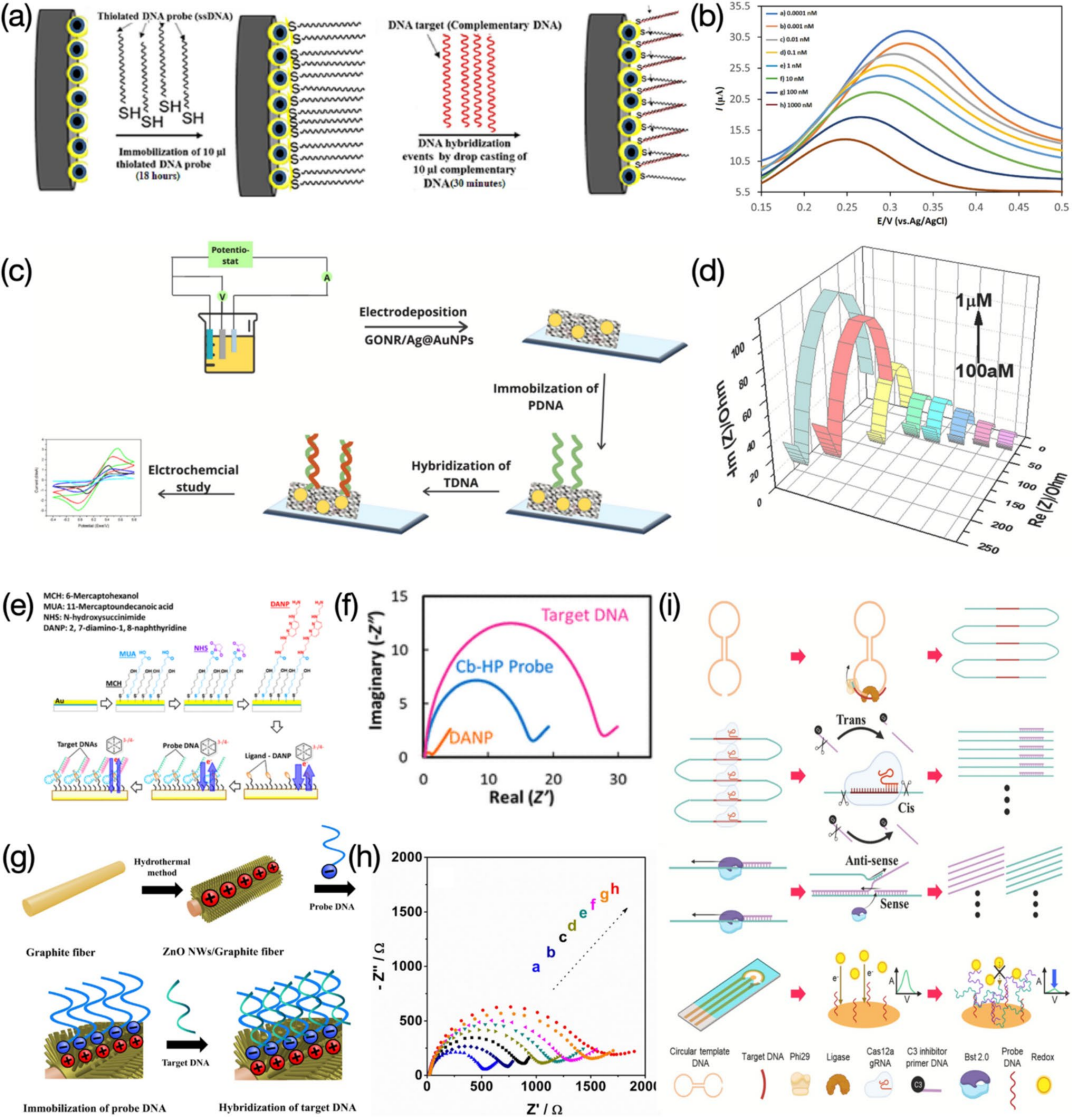
Functionalized electrode-based sensors used for electrochemical detection of DNA, where redox ion pairs are usually suffered from steric hinderance and negative-negative charge repulsion: **a** SPCE coated with Fe_3_O_4_-Au core shell nanocomposites used for detection of HPV-16. Image reprinted with permission from Rasouli et al. (2023); **b** DPV results with concentrations of HPV-16 ranging from 0.1 to 10^6^ pM. Image reprinted with permission from Rasouli et al. (2023); **c** ITO electrode attached with GONR/Ag@AuNPs for determination of HPV-16. Image reprinted with permission from Pareek et al. (2023); **d** EIS measurements with concentrations of HPV-16 ranging from 10^–4^ to 10^6^ pM. Image reprinted with permission from Pareek et al. (2023); **e** On-demand gold-based EIS sensor functionalized with MCH, MUA, NHS, and DANP for detection of target r60 DNA. Image reprinted with permission from Han et al. (2023); **f** Nyquist plot revealing impedance increased after Cb-HP probe sequence attachment and target DNA hybridization. Image reprinted with permission from Han et al. (2023); **g** Graphite fiber wrapped with ZnO NWs for monitoring of target DNA sequences. Image reprinted with permission from Zhang et al. (2019); **h** EIS measurements with concentrations of target DNA sequence ranging from 0.01 to 10^5^ pM. Image reprinted with permission from Zhang et al. (2019); **i** CRISPR-Cas 12a dependent HRCA in combination with electrochemical sensing system for detection of KRAS G12D. Image reprinted with permission from You et al. (2023)

Rasouli et al., created an electrochemical DNA nanosensor for sensitive detection of human papillomavirus type 16 (HPV-16), aiming to provide a reliable sensing platform for early warning of cervical cancer (Fig. 5a) (Rasouli et al. 2023). In this case, the resistance against the charge transfer of [Fe(CN)_6_]^3−/4−^ consists of two parts: one is the intense electrostatic repulsion between the redox couple of [Fe(CN)_6_]^3−/4−^ and the hybridized probe-target DNA complex, the other is the steric hinderance caused by the probe-target DNA hybridization. The sensor provided a linear response towards the concentration of target genes of HPV-16 from 0.1 to 10^6^ pM (Fig. 5b), along with a LOD of 100 pM. Selectivity analysis was performed by comparing the peak current value of DNA probe control, non-complementary, three-base mismatch, one-base mismatch, and complementary DNA. Results revealed the sensor presented less difference in the peak current between the detection of complementary DNA and mismatch sequences. No reproducibility and real sample testing were presented in their work. However, these results could be further improved by another HPV-16 sensor made by Pareek et al. (Fig. 5c), both LSV and EIS characterizations were performed, reaching a wider dynamic range of 10^–4^ ~ 10^6^ pM (Fig. 5d) and a LOD of 10^–4^ pM (Pareek et al. 2023). The selectivity showed the highest impedance against the target DNA but also presented non-negligible responses after hybridization with non-complementary sequences. Two concentrations of HPV-16 were spiked into real samples in this work to evaluate the feasibility for practical usage, and reasonable recovery values were obtained. The sensors fabricated by Rasouli et al. and Pareek et al., were also compared with other recently reported electrochemical biosensors used for detection of HPV, showing a broader dynamic range and a competitive LOD. Han et al. reported an on-demand EIS sensor for detection of the r60 DNA (Fig. 5e, f) (Han et al. 2023). The use of mixed SAMs containing MCH and MUA aimed to form a dense and uniform layer for connection with organic linkers (e.g., NHS coupled with DANP). DNA probes featuring hairpin structures contain the cytosine bulge that can covalently bind to the ligand of DANP. The charge transfer resistance (R_ct_) increased as more target r60 strands were captured, with linear response shown in a range of 10^4^ ~ 10^7^ and a LOD of 10^4^ pM. Zhang et al. developed a DNA nanosensor where the growth of ZnO NWs on the graphite fiber surface was achieved via a hydrothermal method (Fig. 5g) (Zhang et al. 2019). The EIS was used to assess the concentration of target DNA ranging from 0.01 ~ 10^5^ pM (Fig. 5h). Increased impedance signals were observed as higher concentrations of target DNA were introduced. After the demonstration with good signal reproducibility and stability, the selectivity of the sensor was also assessed by testing non-complementary DNA (non-cDNA), single-mismatched DNA, and cDNA, all at same concentration of 10 pM. The highest resistance of charge transfer (i.e., 250 Ω) against the redox ion pair of [Fe(CN)_6_]^3−/4−^ was recorded as the target cDNA was added, while the hybridization with single-mismatched DNA resulted in a lower resistance of 125 Ω, and the case of the non-cDNA provided with almost no resistance. The decrease of resistance is accordance with the synergistic effect of steric hinderance and negatively charged repulsion. Moreover, the electrode after the probe DNA immobilization presented strong reusability of up to ten cycles. Compared with LODs shown in other works, a value of 3.3 × 10^–3^ pM yielded from the sensor is considered as a highly competitive LOD, which implies that the impedimetric methods can offer ultrasensitive detection over other electrochemical techniques particularly with redox couples suffered from dual hinderance. You et al., introduced the CRISPR-Cas 12a dependent hyperbranched rolling circle amplification (HRCA) into the sensing system for the electrochemical detection of Kristen rat sarcoma viral oncogene homolog (KRAS G12D) (Fig. 5i) (You et al. 2023). A variety of bioactive enzymes including ligase and polymerases (e.g., phi29 and Bst 2.0) were used contributing to amplification of target sequences from both sense and anti-sense direction. The resulting target strands then bound to the probe DNA, leading to increased spatial resistance and more intense repulsion force against the redox couple. The sensor enabled a wide range of detection ranging from 10^–6^ to 1 pM, along with a LOD of 10^–5^ pM. The good selectivity of the sensor was demonstrated by detecting the KRAS G12D mixed with other DNA interferents, and only the target owned the highest current over 300 μA.

**Fig. 6:**
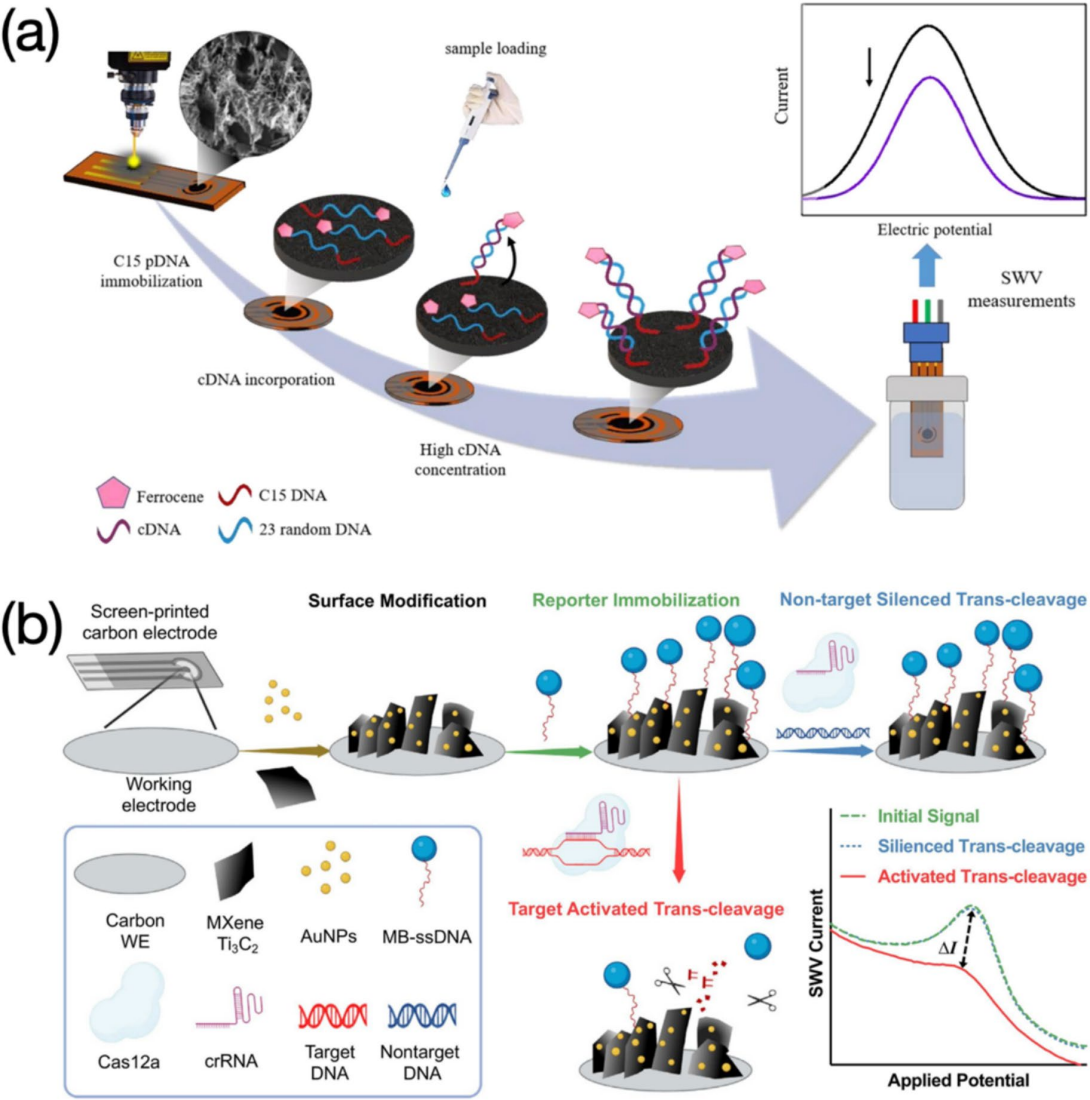
Functionalized electrode-based sensors used for electrochemical assessment of DNA, from which signal variations are sourced from increased distance between electrochemical tags and electron transfer interface: **a** LIG based electrochemical sensor for detection of cDNA. Image reprinted with permission from Bahri et al. (2023); **b** SPCE modified with MXene Ti_3_C_2_ and AuNPs for target DNA determination. Amount of MB molecules attached at electrode surface are controlled via target activated trans-cleavage, thus causing different electrochemical signals. Image reprinted with permission from Duan et al. (2023)

Hybridization between complementary DNA (cDNA) and probe DNA (pDNA) molecules was quantified by means of a LIG based electrochemical sensor (Fig. 6a) (Bahri et al. 2023). Square wave voltammogram showed a weakened variation of peak current after series of concentrations of cDNA from 0.1 to 100,000 pM were measured. Ferrocene (Fc) was used as an electrochemical tag aiming to yield obvious current signals. Decreased peak currents are recorded due to Fc molecules lifted from the electrode surface after the hybridization, extending the distance of electron transfer towards the electrode. A femto-level LOD was eventually obtained using this sensing strategy. Non-complementary DNA (ncDNA) and several mismatch DNA strands with a concentration of 100,000 pM were involved to evaluate the selectivity of the sensor, yielding negligible signals compared to that of the cDNA. Good reproducibility was investigated by the determination of cDNA at 1,000 pM on three individual sensors. The sensor was also tested in tenfold diluted human serum, verifying a good adaptability in real sample measurement. Based on a similar sensing mechanism, Duan et al., provided an electrochemical DNA sensor integrated with a biological component of CRISPR-Cas 12a for highly sensitive detection of HPV-18 (Fig. 6b) (Duan et al. 2023). Serving as a signal indicator, methylene blue (MB) molecules are confined near the electrode surface since non-target DNA sequences failed to induce the trans-cleavage activity, leaving obvious current signals. The CRISPR-Cas 12a along with the programming CRISPR-RNA (crRNA) triggers the trans-cleavage activity once target DNA sequences hybridize with the MB conjugated ssDNA, causing reduced peak currents. The proposed sensor exhibited excellent selectivity for both HPV-16 and HPV-18, while had no response towards other HPV subtypes. Based on the good performance in the real urine sample testing, human immunoglobulin G (IgG) and bovine serum albumin (BSA) were tested in both urinal and standard buffered samples to evaluate the sensor selectivity for non-target proteins, with negligible differences observed in current signals. Beyond a highly competitive detection range of 10 ~ 500,000 pM and a LOD of 1.95 pM, the sensor also equipped with good stability and less response time, compared to other DNA detections based on CRISPR-Cas 12a powered electrochemical sensors.

**Fig. 7:**
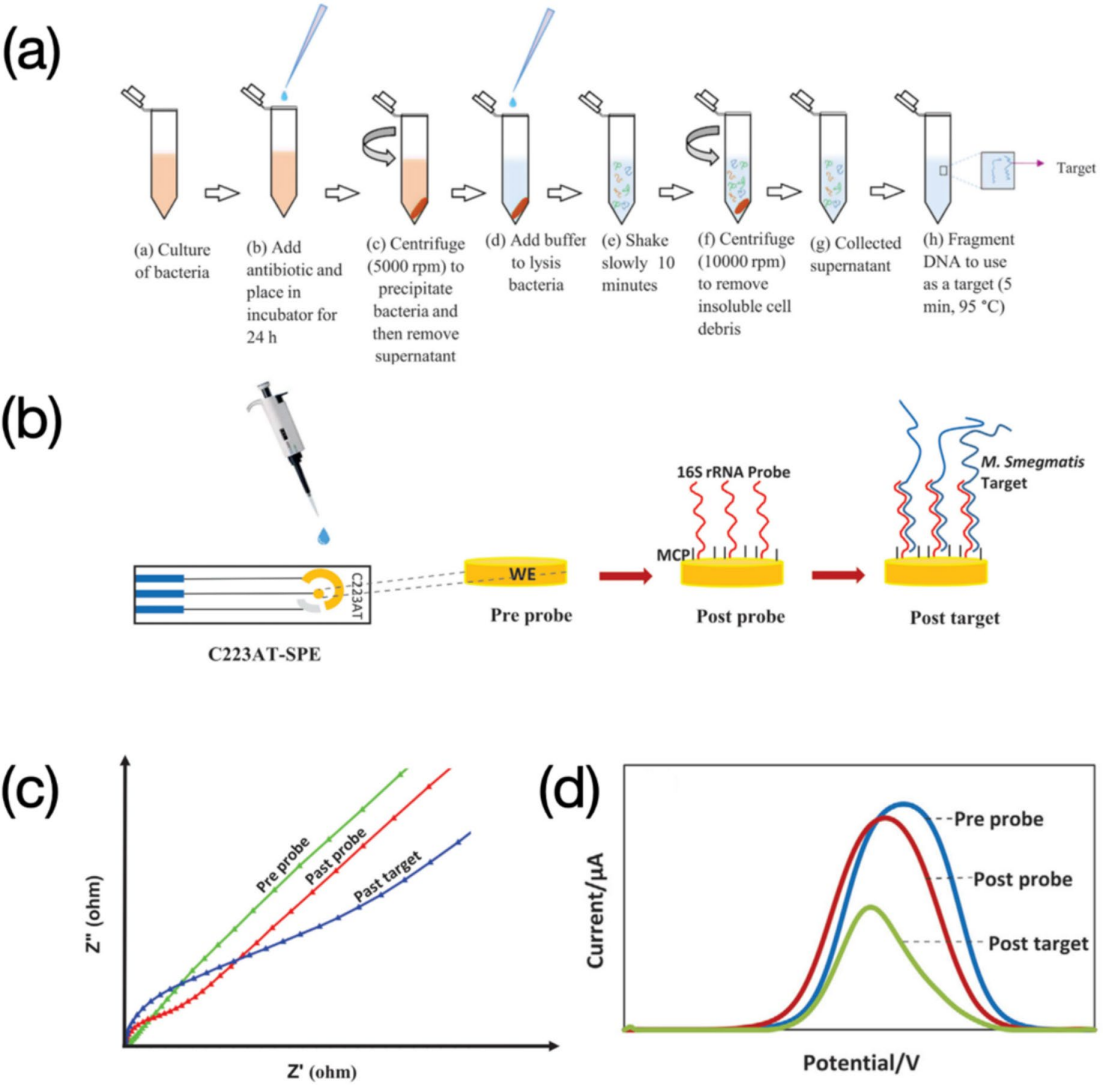
16S rRNA probe functionalized sensors for electrochemical detection of *M. Smegmatis* DNA target: **a** Preparation of *M. Smegmatis* DNA target from bacteria culture. Image reprinted with permission from Güzel et al. (2021); **b** Detection of *M. Smegmatis* DNA target using C223AT-SPE, including immobilization of 16S rRNA probe and *M. Smegmatis* DNA target hybridization. Image reprinted with permission from Güzel et al. (2021); **c** Positively varied EIS responses after a sensing process. Image reprinted with permission from Güzel et al. (2021); **d** Negatively varied SWV responses after a sensing process. Image reprinted with permission from Güzel et al. (2021)

DNA probes used are mostly ssDNA strands. However, other biological sequences complementary to DNA targets such as RNA are also applied for electrochemical detection of DNA. Güzel et al., developed an effective electrochemical sensor for detection of antibiotic susceptibility, where the surface of the C223AT-SPE was uniformly functionalized with 16S rRNA probes for recognizing *M. Smegmatis* DNA targets (Fig. 7b) (Güzel et al. 2021). The backfilling solution containing 3-mercapto-1-propanol (MCP) and tris(2-carboxyethyl) phosphine (TCEP), along with the probe solution was prepared for the formation of SAM. The targeted fragment DNA was obtained after the culture of bacteria coupled with a series of centrifugation (Fig. 7a). Two different electrochemical techniques (i.e., EIS and SWV) were applied to achieve the detection of the *M. Smegmatis* DNA target. Current values in the SWV measurement negatively varied after the introduction of DNA targets (Fig. 7d), which was in accordance with the mechanism that increased electrostatic resistance or steric hinderance could debilitate the electron transfer of redox couples. While EIS results presented the trend of variation in an opposite behavior (Fig. 7c). The impedance drastically increased after stages from “pre probe” and “past probe” to “past target”, implying the positive correlation between electrochemical signals and DNA analyte concentrations. More studies revealing this positive correlation can be found below.

### Positive correlation between electrochemical responses and target DNA concentrations

**Fig. 8:**
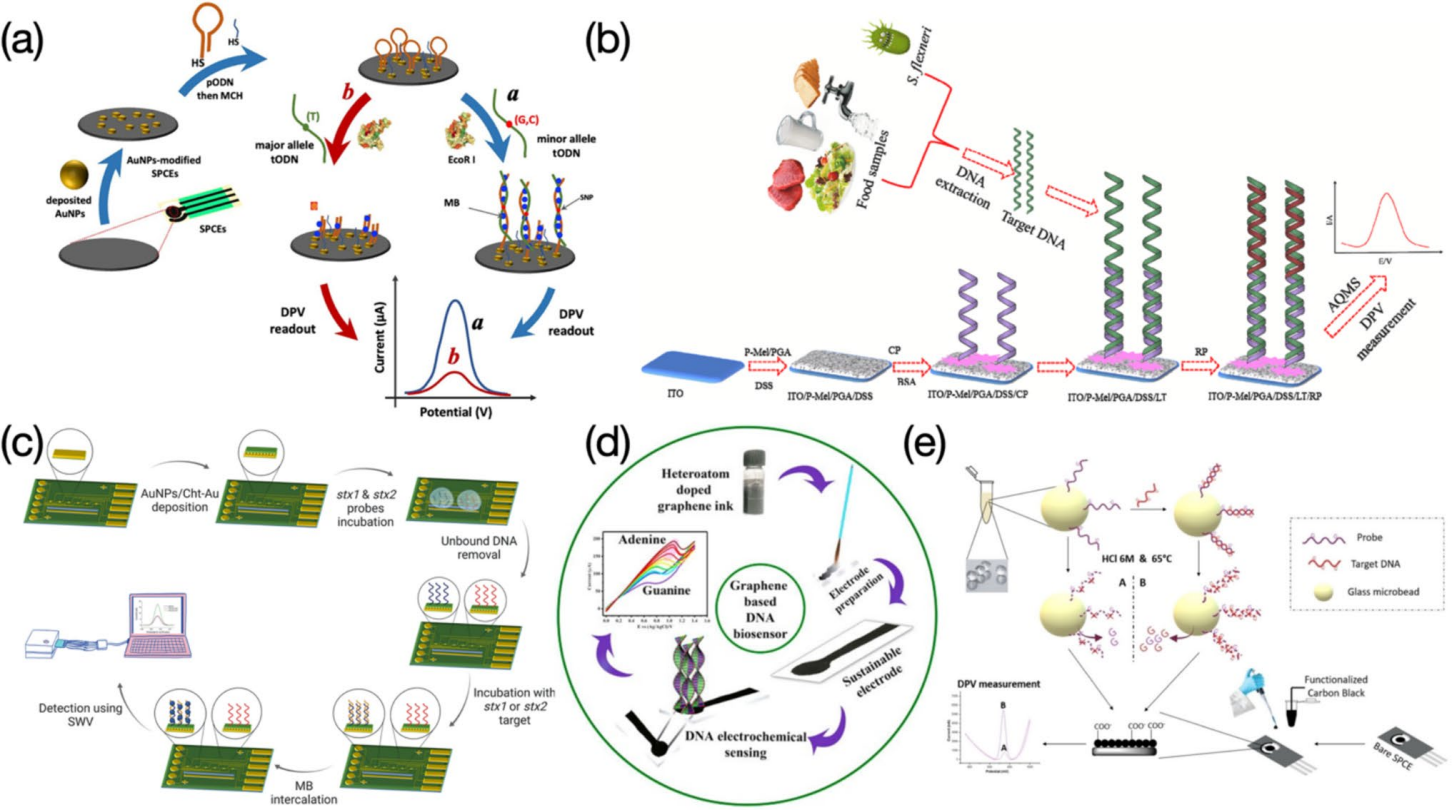
Schematic illustration of electrochemical sensors used for assessing DNA molecules, where electrochemical results are caused by electron transfer promotion in presence of signal indicators intercalated in double-stranded structures: **a** EcoRI assisted electrochemical bio-platform prepared for determination of polymorphisms in IL6 and TGFβ1 genes. Image reprinted with permission from Meftah et al. (2023); **b** Flexible ITO based nanosensor used for monitoring of *S. flexneri* genes. Image reprinted with permission from Ali et al. (2023); **c** Multiplex silicon supported DNA nanosensor for simultaneous detection of *stx1* and *stx2* genes. Image reprinted with permission from Wasiewska et al. (2023); **d** Paper-based electrochemical sensor with nanostructures of N-doped graphene used for nucleobase detection of A and G from calf thymus DNA. Image reprinted with permission from Jagannathan et al. (2023); **e** f-CB/SPE based electrochemical sensor used for determination of A and G released from DNA hydrolysis. Image reprinted with permission from Aamri et al. (2023)

Electrochemical responses recorded have a positive correlation with target DNA concentrations, which are mainly attributed to the use of redox tags intercalated in probe-target DNA complex. A restriction endonuclease EcoRI assisted bio-platform prepared by Meftah et al. was used to determine polymorphisms in both interleukin-6 (IL6) and transforming growth factor β1 (TGFβ1) genes from patients suffered from ovarian cancer (Fig. 8a) (Meftah et al. 2023). MCH acting as a blocking agent was applied to the electrode surface to avoid any non-specific adsorption at active sites. MB molecules used as a signal indicator promised an increased peak current once they intercalated into the double-stranded oligonucleotide sequences (ODN). The sensor presented wide linear ranges of 0.1 ~ 100,000 and 0.05 ~ 100 pM, along with LODs of 0.048 and 0.017 pM, for IL6 and TGFβ1, respectively. The selectivity towards two genes was assessed by mixing with their 1-, 2- mismatch, and non-complementary sequences and only target IL6 and TGFβ1 genes feedbacked a significant high response even the concentration used was 30 times lower than those of rest interferents. Moreover, the sensor was utilized in real samples containing TT, TG/TC, and GG/CC genotypes, and achieved good results on the discrimination in heterozygous and homozygous genes. Sensing performances regarding the proposed sensor were compared with those from other reported electrochemical nanosensors for DNA polymorephism detection. However, target genes and restriction endonuclease applied were not very consistent as the current study, which were not able to provide convincible results in the literature comparison. Ali et al. developed an electrochemical DNA nanosensor for monitoring genes extracted from *Shigella flexneri* (*S. flexneri*) bacteria (Fig. 8b) (Ali et al. 2023). Capture probes (CP) were introduced forming a uniform coverage for immobilization of linear targets (LT). Report probes (RP) were also involved in hybridizing with the LT. The anthraquinone-2-sulfonic acid monohydrate sodium salt (AQMS) played as a role of signal indicator and was applied in the detection, reaching a positive correlation between DPV responses and concentrations of the target DNA, probably due to the accommodation of the AQMS in the LT-RP double-stranded structure. The sensor presented an extremely wide detection range from 10^–9^ to 10^6^ pM, along with an ultralow LOD of 7.4 × 10^–10^ pM. The developed nanodevice can be utilized for determination of other target sequences by easily changing the CP, LT, and RP, revealing a strong application versatility. The largest current response was produced by the hybridization with LT, compared with those yielded from mismatched and non-complementary LT sequences. In detection of pathogenic bacteria, *S. flexneri* series exhibited higher current signals over other categories. These demonstrate high selectivity of the sensor for both DNA and bacteria detection. Moreover, the eminent stability makes the sensor still provide measurable signals even after very long-term storage (e.g., up to 8 weeks). The reusability was also tested by breaking the DNA duplex and rebinding with the LT several times, and results showed that not much apparent signal decreasing was recorded within 5 ~ 6 regeneration cycles. The target *S. flexneri* DNA added real food samples were eventually analyzed, with recovery values ranging from 90 ~ 103%, evidencing good practicability of the sensor. Wasiewska et al., prepared a multiplex silicon supported DNA nanoelectrode for simultaneous determination of *stx1* and *stx2* genes from the Shiga toxin-producing *E. coli* (STEC) (Fig. 8c) (Wasiewska et al. 2023). The robust functional layer with very good conductivity was achieved by electrodepositing AuNPs and the chitosan-gold nanocomposite (Cht-Au) on the gold interdigitated electrode (Au-IDE), providing biocompatible circumstance for attachment of probe DNA sequences (Wasiewska et al. 2022). MB molecules increasingly intercalated into the probe-target DNA duplex as concentrations detected from 10^–7^ to 10^–2^ pM and 10^–7^ to 10^–1^ pM, for *stx1* and *stx2* genes respectively, causing increase of square wave voltammetry (SWV) responses and an ultralow LOD of 10^–7^ pM. The nanosensor was also tested after simultaneously introducing two types of probe sequences complementary to target genes, showing strong discrimination ability particularly with presence of the MB. In comparison to previously reported DNA sensors used for detection of genes from the STEC, the sensor presented the lowest LOD. However, target genes compared were not always *stx1* and *stx2*. Jagannathan et al., developed a type of paper-based electrodes functionalized with nanostructures of N-, P-, and S- doped graphene and used to detect calf thymus DNA (Jagannathan et al. 2023). The cathodic cyclic voltammetry was applied throughout the detection towards guanine (G) and adenine (A). Results yielded from the N-doped graphene electrode present characterizable current signals for both G and A, compared with other heteroatom doped (e.g., P-, S- doped) or untreated ones that can only detect G with less discrimination (Fig. 8d). The sensor eventually presented a concentration range of 4 × 10^5^ ~ 4 × 10^6^ pM, and a LOD of 1.5 × 10^6^ pM for detection of G, and a concentration range of 4.4 × 10^5^ ~ 4.4 × 10^6^ pM, and a LOD of 2.38 × 10^6^ pM for detection of A. Aamri et al., performed a similar work of determining both G and A released from DNA hydrolysis based on a f-CB assisted electrochemical sensor (Fig. 8e) (Aamri et al. 2023). Glass microbeads were used for immobilization of probe DNA strands or probe-target DNA duplexes. The reaction was allowed to be carried out in a tube, from which the resulting solution was drop-casted onto the f-CB layered electrode surface, followed by a DPV measurement to evaluate the amount of released G or A molecules. The proposed DNA nanosensor exhibited good selectivity towards target strands, since electrochemical responses of the target are remarkably higher than those of the probe only or non-complementary sequences. The repeatability was also assessed by testing three equally prepared sensors incubated with the target of microRNA-21 at different concentrations, yielding relatively consistent results with a RSD of 5.6%. In comparison to the work of Jagannathan et al., the sensor presented in this study enables a slightly wider detection range towards both G and A, although LOD values obtained have not been apparently improved. Additionally, there are many reports on determination of G and A, or thymine (T), based on various electrochemical sensors, yielding more broad ranges of determination along with several order-of-magnitude lower LODs, compared with the current two works, indicating further development upon the sensor is needed to improve sensing performances for detection of DNA bases.

**Fig. 9:**
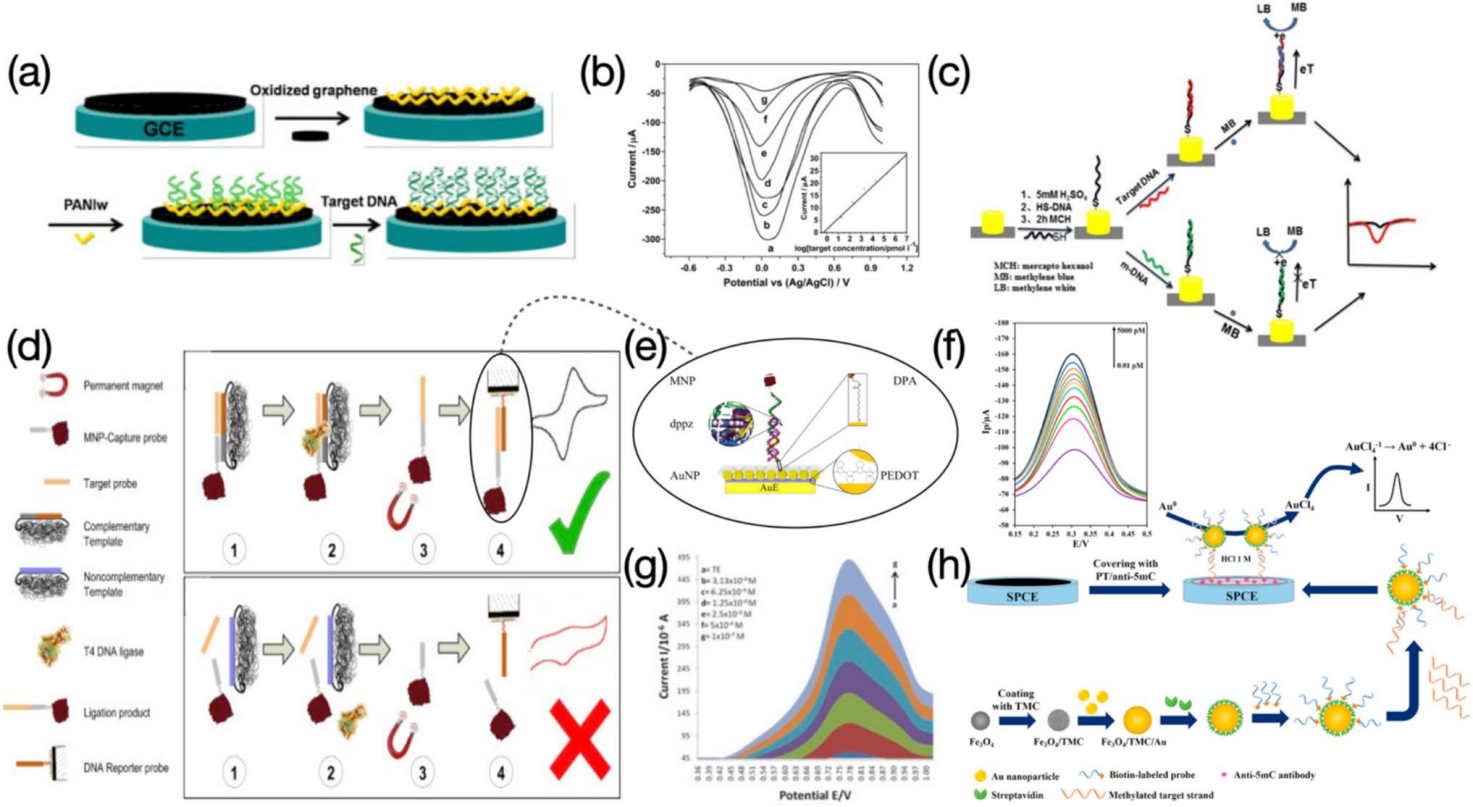
**a** GCE modified with GO and PANIws used for detection of target DNA molecules. Image reprinted with permission from Bo et al. (2011); **b** DPV responses in relation to different concentrations of target DNA from g to a representing a range of 2.12 ~ 2.12 × 10^6^ pM. Image reprinted with permission from Bo et al. (2011); **c** AuNWE covered with MCH and thiolated DNA probes for electrochemical analysis of DNA. Image reprinted with permission from Hua et al. (2018); **d** T4 DNA ligase involved DNA sensing mechanism, along with presence of MNP for target sequence enrichment. Image reprinted with permission from Hushiarian et al. (2015); **e** Gold-based biosensor coupled with PEDOT-PSS, AuNPs, and DPA used for sensitive detection of genomic DNA, an image with more details revealing successful hybridization. Image reprinted with permission from Hushiarian et al. (2015); **f** DPV responses in relation to different concentrations of methylated target strands ranging from 0.01 to 5,000 pM. Image reprinted with permission from Daneshpour et al. (2016); **g** DPV responses in relation to different concentrations of target probes ranging from 3,130 to 10.^5^ pM (a to g). Image reprinted with permission from Hushiarian et al. (2015); **h** SPCE covered with PT and anti-5mC antibody used for immobilization of methylated target strands. Nanocomposites of Fe3O4/TMC/Au/streptavidin hybridized with biotin-labeled probe sequences forming a “sandwich” sensing strategy after they recognize methylated target strands. Image reprinted with permission from Daneshpour et al. (2016)

In the work of Bo et al. (Bo et al. 2011), the surface of oxidized graphene coupled with PANIws was elaborately fabricated to provide an adequate, biocompatible, and highly conductive room for immobilization of probe sequences (Fig. 9a). A series of linear DPV signals were observed after a wide dynamic range of 2.12 ~ 2.12 × 10^6^ pM was tested, where reduction peaks became stronger with the increment of target DNA concentration (Fig. 9b). Daunomycin was used as the electroactive signal indicator, of which the reduction was achieved via transfer of electrons after the formation of probe-target double helix structure. The proposed nanosensor demonstrated that the electrode surface modified with probe-target DNA duplexes presented the highest current response in the presence of daunomycin, compared to cases of base mismatched sequences and noncomplementary DNA. Other than the low detection limit of 0.325 pM, the reproducibility and repeatability were also studied on seven sensors, with a small RSD of 1.15%. The storability was evaluated by placing the electrode in 0.1 M phosphate buffered saline (PBS), pH 7.3, at 4 °C for over seven days, and current signals with a slight decrease were observed. The proposed sensor owns a wider linear range coupled with a lower LOD in comparison to values shown in other reports. Hua et al., allowed MB molecules to undergo a reduction into methylene white (LB) on a single gold nanowire electrode (AuNWE), serving as indicator of SWV signals for electrochemical analysis of DNA (Fig. 9c). (Hua et al. 2018) Probe DNA sequences were fixed on the sensor surface via the gold-thiol bonding, followed by the hybridization with target DNA strands to enable MB molecules to intercalate into the probe-target DNA duplex through the intense electrostatic effect (Dai et al. 2012; Yau et al. 2003). However, the mismatched hybridization led to the failure of MB intercalation, thus providing no reduction signal. The nanosensor presented a linear response over concentrations ranging from 10^–3^ ~ 10^4^ pM, yielding a detection limit of 4.8 × 10^–4^ pM. The LOD was three orders of magnitude lower than that presented in the work of Bo et al. (Bo et al. 2011), two orders of magnitude lower than that obtained from the GCE modified with AuNPs, reduced graphene oxide (rGO), and Adriamycin (Zhang and Jiang 2012), as well as lower than results in other MB-assisted electrochemical sensors. An even lower LOD of 1.1 × 10^–4^ pM was produced by Wang et al. (Wang et al. 2014), through a simple GCE covered with electropolymerized Eriochrome Black T (pEBT). Nevertheless, the detection range was very narrow compared with this work (i.e., 10^–3^ ~ 10^4^ pM). In addition, the sensor stability was also tested, however, current signals recorded were with non-negligible decreases only after 6 days. A gold-based biosensor modified with poly(3,4-ethyllenedioxythiophen)-poly(styrenesulfonate) (PEDOT-PSS) and AuNPs were constructed for detection of the genomic DNA in white rot fungus, *Ganoderma bonienese* (Fig. 9e) (Hushiarian et al. 2015). Initially, MNP-Capture probes bound to the complementary template and intended to link with the target DNA sequence in the presence of T4 DNA ligase, yielding extended DNA strands (35-mer). The 35-mer DNA fragment then dissociated from the template after a heating treatment and was isolated from other undesired DNA sequences under an applied magnetic field, followed by hybridization with DNA reporter probes that were immobilized on the electrode surface by means of a self-assembled DPA monolayer (Fig. 9d). While the use of non-complementary template resulted in the failure of probe-target hybridization, leaving MNP-Capture probes only (Fig. 9d). Ru(dppz), another redox complex, was applied in this work to produce sensitive signals after they inserted into double-stranded structures. A positive correlation between DPV signals and target DNA concentrations was observed (Fig. 9g), yielding a LOD of 0.054 pM. This work employs magnetic nanoparticles and DNA ligase for separation of target sequences from non-specific substances, which presents great significance in the detection of genomic DNA with various types, although the value of detection limit is not as low as that shown in their previously reported paper (Dutse et al. 2014). A “sandwich” sensing strategy for the electrochemical assessment of RAS association domain family (RASSF) 1 A tumor suppressor DNA methylation was reported by Daneshpour et al., where the electrode was functionalized with polythiophene (PT) and the anti-5-methylcytosin (anti-5mC) monoclonal antibody (Fig. 9h) (Daneshpour et al. 2016). Fe_3_O_4_ nanospheres coated with N-trimethyl chitosan (TMC) are preferable for attachment of AuNPs, forming Fe_3_O_4_/TMC/Au nanocomposites to anchor streptavidin molecules. Biotin-labeled probe sequences subsequently grew at the surface of the nanosphere owing to the strong affinity between streptavidin and biotin. Methylated target DNA capable of being captured by the anti-5mC antibody bound to probe sequences dragging the Fe_3_O_4_/TMC/Au nanocomposite nearby the electrode surface, forming a “sandwich” configuration for electron transfer of AuNPs (Fig. 9h). The DPV characterization evidenced that the genosensor had an extremely wide concentration range of methylated DNA ranging from 0.01 to 5,000 pM. The reduction peak current varied in a positive correlation with the concentration of target sequences (Fig. 9f). A reasonable explanation is the more methylated target DNA strands are introduced, the more Fe_3_O_4_/TMC/Au nanocomposites land at the electrode surface, causing reduction peak current signals with higher intensity as increased amount of gold ions are reduced into the elemental gold. An estimated LOD of 0.002 pM was presented in their work and was proved lower than LODs yielded from other relative studies. The specificity of the sensor was evaluated by analyzing signals of methylated sequences, unmethylated sequences, and sheared genomic DNA, at concentrations of 500 and 1,000 pM, and only the methylated sequence provided measurable responses. The excellent signal reproducibility and repeatable electrode fabrication make the work highly useful in many practical applications. The proposed sensor was tested in real plasma solutions containing five different concentrations of the methylated DNA, reaching a good recovery range with a relatively small RSD. Additionally, the sensor also displayed acceptable long-term stability with a slight decrease of peak reduction current obtained from electrodes already stored for 1 to 8 weeks at 4 °C.

### Electrochemical assessment of DNA using nanopore-based sensors

Nanopores, another class of prevalently advocated sensing devices, have been widely developed for high-resolution assessment of DNA molecules. In a scheme of nanopore-based sensing, ssDNA molecules thread through a nano-dimensional channel under an applied potential, causing blockage of ions and thus yielding current signals varied with time. By virtue of rapid data recording, excellent precision, and cost-effectiveness, nanopores have been employed in characterization of various biological analytes such as ssDNA or double-stranded DNA (dsDNA) sequences, aptamers (i.e., short-length of ssDNA or RNA), peptides, proteins, and DNA-peptide conjugates (Venkatesan et al. 2009). DNA translocation events are usually measured through ionic current when a DNA sequence threading through a nanopore. Other measuring protocols such as tunnelling current is also used in nanopore-based assessments of DNA, although there is no successful report in identifying DNA bases using tunneling current (Ivanov et al. 2011). Nanopores can be largely classified into two subtypes namely biological nanopores and solid-state nanopores. Biological nanopores can be made through a variety of materials including toxins (Sarangi and Basu 2018), porins (Wang et al. 2021), pore-forming proteins (Parker and Feil 2005), and substrate-specific channels such as DNA domains (Langecker et al. 2012). However, the diameter of channels within most of them is too large to detect DNA. Some of the porins where the size of constriction zone can be down to 1 ~ 2 nm are considered as terrific nanopore candidates for DNA sequencing. Therefore, this review mainly focuses on the porin-based nanopores rather than other biological counterparts. Also, breakthroughs particularly for ssDNA sequencing at individual-nucleobase resolution facilitate porin-based nanopores to be developed into more promising nanodevices suitable for high-throughput biological analyses or medical diagnoses. As another crucial nanopore subtype, solid-state nanopores are discussed at the later stage of the review.

### Porin-based nanopores for DNA sequencing

Porin-based nanopores, also named transmembrane proteins, are the initial elaboration of scientific groups aiming for continuous DNA sequencing. The double-layer phospholipid membrane prefers to cover onto a chip surface containing an artificial aperture normally with a diameter of several micrometers. Driven by the electrophoretic force (i.e., cathode in the *cis-* compartment and anode in the *trans-* compartment), transmembrane proteins then move towards the membrane and ultimately embed within the membrane. Blockages against ions are created while biological molecules (e.g., DNA) pass through the pore, which are recorded via a real-time signal transformation setup. Current responses with different magnitudes, shapes, and dwell times, revealing information of tested sequences, are deemed as evaluation criteria for DNA sequencing (Deamer et al. 2016). An excellent overview reported by Crnkovic ´ et al., mainly focuses on commonly used porin-based nanopores, outlining information about geometric dimensions regarding proteinaceous nanochannels (Crnković et al. 2021). Among them, α-hemolysin (α-HL) and *Mycobacterium smegmatis* porin A (MspA) are commonly reported porin-based nanopores, which can be massively produced through a mature process of *Escherichia coli* (*E. coli*) induced protein expression. Although it is complicated and time-consuming, this method ensures porin-based nanopores, prepared from batch to batch, are with an identical dimension at a molecular level. Up to now, porin-based nanopores have been developed into various mutated forms catering for high-resolution DNA and protein sequencing.

**Fig. 10:**
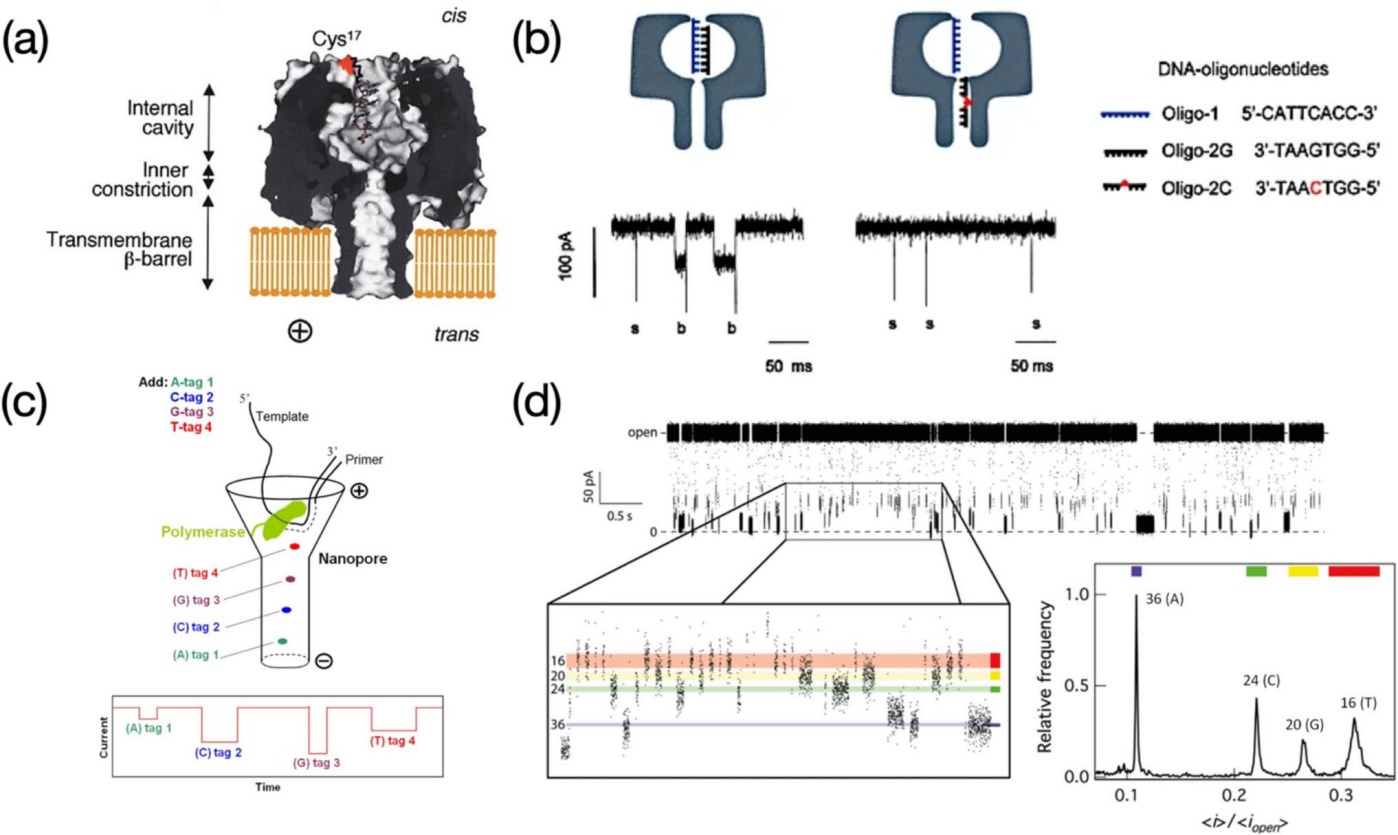
**a** α-HL nanopore with a DNA oligonucleotide anchored at mutated site of Cys 17. Image reprinted with permission from Howorka et al. (2001); **b** Probe oligo-1 hybridized with complementary oligo-2G sequences or one-base mutated oligo-2C, leading to different threading signals. Image reprinted with permission from Howorka et al. (2001); **c** Schematic illustration of tags threading through a DNA polymerase immobilized α-HL nanopore, along with different current blockages revealing four nucleobases. Image reprinted with permission from Kumar et al. (2012); **d** Statistical study on tags passing through nanopore to achieve discrimination of bases. Image reprinted with permission from Kumar et al. (2012)

DNA oligonucleotides with different sequences were successfully discriminated through the α-HL nanopore with a probe DNA sequence anchored at the Cys^17^ mutation site (Fig. 10a) (Howorka et al. 2001). Target sequences hybridizing with the probe caused physical hindrance against ion flows, yielding signals with a lowered current level. They were subsequently torn apart from its complementary sequence due to the electric field force and threaded through the β-barrel region of the engineered α-HL nanopore, causing unique downward spike-shaped signals. Nevertheless, mismatched sequences (e.g., single-base altered DNA) directly passed through the pore without any obstruction owing to the absence of base-to-base hybridization, presenting downward spikes merely (Fig. 10b). This strategy is useful in recognizing target DNA sequences in hodgepodge DNA samples but may not be very suitable for continuous DNA sequencing as lack of a component serving as a ‘brake’ for rapid DNA translocation. Moreover, the protein mutation involved in this work makes the nanopore fabrication more complicated and increases time cost. Facing the challenge of high translocation speed of DNA, some enzymatic components specific to DNA strands such as DNA polymerases or helicases were introduced to slow down the translocation event. Nanopore sensing integrated with DNA polymerases, as so-called sequencing by synthesis (SBS), has been regarded as one of routes to DNA sequencing at a single-molecule level. Tagged nucleotides are assembled into a DNA strand in the presence of DNA polymerases, while tags indicative of different nucleobases dissociates from nucleotides and are sequentially move through the nanopore, leading to base-corresponding current signals (Fig. 10c). In Kumar’s work, detectable tags containing a small molecule of coumarin coupled to polyethylene glycol (PEG) in different lengths including PEG_16_, PEG_20_, PEG_24_, and PEG_36_, were prepared reflecting nucleobases of thymine (T), guanine (G), cytosine (C), and adenine (A), respectively (Kumar et al. 2012). Current drops with various magnitudes caused by blockage of tags were statistically recorded, revealing information of four nucleobases (Fig. 10d). The proposed method verifies an indirect DNA sequencing relying on base-related tags. However, the preparation of tags lengthens the experimental procedure before the nanopore sensing. According to results, signals with respect to bases of T, G, and C present a close magnitude except the base of A, which to some extent, may increase errors in base identification. A possible interpretation is use of PEG chains with similar lengths (i.e., PEG_16_, PEG_20_, and PEG_24_) for indicating bases of T, G, and C, while a relatively longer chain of PEG_36_ represents the base of A. It is more worth noting that current blockages on bases shown in this study are not rigorously consistent with outcomes presented in other works (Stranges et al. 2016; Fuller et al. 2016), as it may be due to different chemical structures or lengths of tags that cause spatial blockages in different forms.

**Fig. 11:**
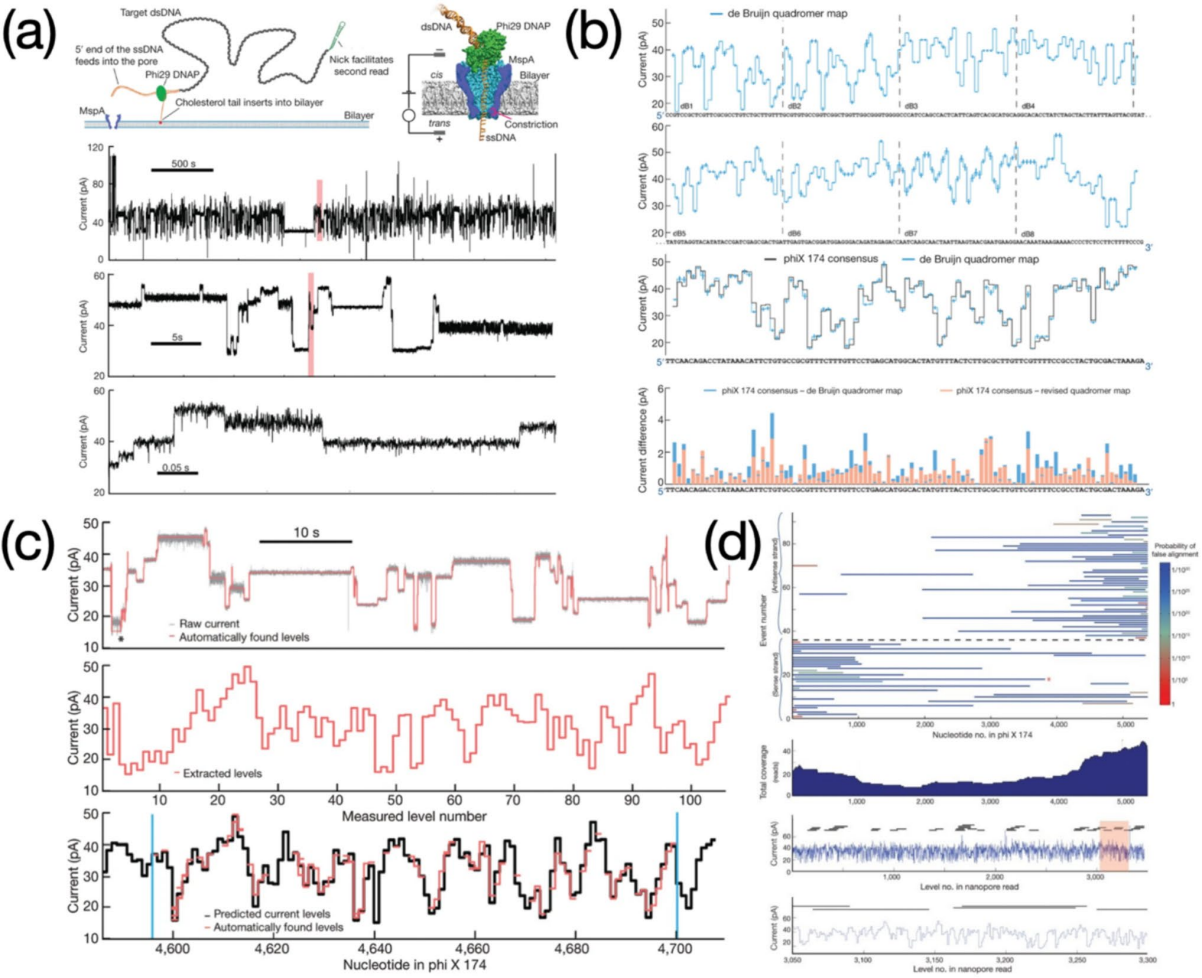
**a** phi29 DNA polymerase assisted DNA sequencing based on MspA nanopore and continuous recording of target DNA sequences. **b** Prediction on current signals of previously unevaluated sequence using de Brujin quadromer map, along with the comparison with phi X 174 consensus; **c** Data processing by means of a level-finding algorithm and alignment between measured current blockages and predicted values; **d** Analysis regarding false alignments based on 91 reads of phi X 174 genomic DNA. Image reprinted with permission from Laszlo et al. (2014)

A sensing ensemble of MspA nanopore integrated with phi29 DNA polymerase (DNAP) was reported to successfully realize a clear and continuous decoding of DNA sequences (Laszlo et al. 2014). Target analytes were elaborately designed into a phi29 DNAP attached short length of ssDNA linking to the target double stranded DNA (dsDNA) molecule coupled with a second adaptor for sequence rereading (Fig. 11a). Complementary sequences attach to the lipid bilayer due to presence of cholesterol ends, aiming for the analyte enrichment. The ssDNA, under the force of electric field, passed through the MspA pore first, dragging the phi29 DNAP and dsDNA towards the pore as well. The phi29 DNAP was then blocked at the *cis-* entrance of the MspA pore owing to the oversized proteinaceous conformation, conducted polymerization at a pace of single nucleobase to reduce the speed of DNA translocation, yielding continuous current signals varied with clear stairs (Fig. 11b). Comparisons in relation to ionic current readouts between the sequence measured and reference values predicted by the de Brujin quadromer map were evaluated (Fig. 11b), along with the analysis of false alignment based on 91 reads of phi X 174 genomic DNA aiming to enhance the reliability of nanopore sequencing (Fig. 11c,d). Next development for the work was to apply a reference sequence to calibrate amplitude of ion current signals for the *de novo* DNA sequencing. Also, some locations within a piece of current signal recording were in the lack of transient pore-threading information probably due to the phi29 DNAP movement or mathematical errors in calculation of the level-defining algorithm (Laszlo et al. 2014). The use of DNA helicases was depicted as a future attempt to significantly improve DNA sequencing because of the monotonous moving nature of helicases upon DNA strands (Laszlo et al. 2014).

### Solid-state nanopores for DNA sequencing

Solid-state nanopores for DNA sequencing can be made using either “top-down” or “bottom-up” methods. “Top-down” methods such as focused ion beam (FIB) and focused electron beam (FEB) allow high-energy particles (e.g., gallium (Miles et al. 2013) and helium (Marshall et al. 2012) ions, or electrons (Chen et al. 2017)) to penetrate the surface of thin film, creating in-plane defects with various geometries. System temperature, beam duration, or intensity are key parameters in the process of beam-induced nanopore fabrication. Altering them or one of them can yield solid-state nanopores with a very small diameter. Interestingly, electron beams acting at 2D material surfaces for a longer duration also result in smaller nanopores. (Li et al. 2001) Reactive ion etching (RIE) applies chemically to reactive plasma (e.g., O_2_ plasma) to engrave treated surfaces, leaving cavities especially for thick wafers (Bai et al. 2014), which is deemed as another effective method for nanopore creation. “Bottom-up” methods, sometimes called self-assembly methods to nanopore fabrication, often enable nanopore-containing films to be constructed based on material building blocks. In contrast with traditional nanopores in silicon nitride substrate, there are also substantial studies focusing on production of hybrid nanopores. They are a class of special solid-state nanopores that are always functionalized with bio-components for overcoming the drawbacks of bare solid-state nanopores including less biocompatibility and difficulty of active site creation (Yusko et al. 2011; Bell et al. 2012). Yusko, et al., created hybrid nanopores of which the surface of silicon nitride nanopore was coated with lipid membrane. Compared with untreated silicon nitride nanopores, the lipid-coated one can allow an individual protein molecule translocating through the channel without clogging (Yusko et al. 2011). Hybrid nanopores have versatile functions, however, the fabrication process may be time-consuming with a higher cost. Moreover, the length of the channel is not radically changed as most of them are still limited by the thick solid substrate, leading to current blockages observed are not caused by a single base or amino acid. Nanopore contraction techniques also belong to “Bottom-up” methods. Among them, metal oxide deposition induced nanopore contractions are realized by introducing an ultrathin layer of metal oxides such as Al_2_O_3_, TiO_2_, and HfO_2_ through the atomic layer deposition (Chen and Liu 2019). However, this technique often leads to an increase of membrane thickness, causing length nanopore channels that are not suitable for high-resolution DNA reading.

**Fig. 12:**
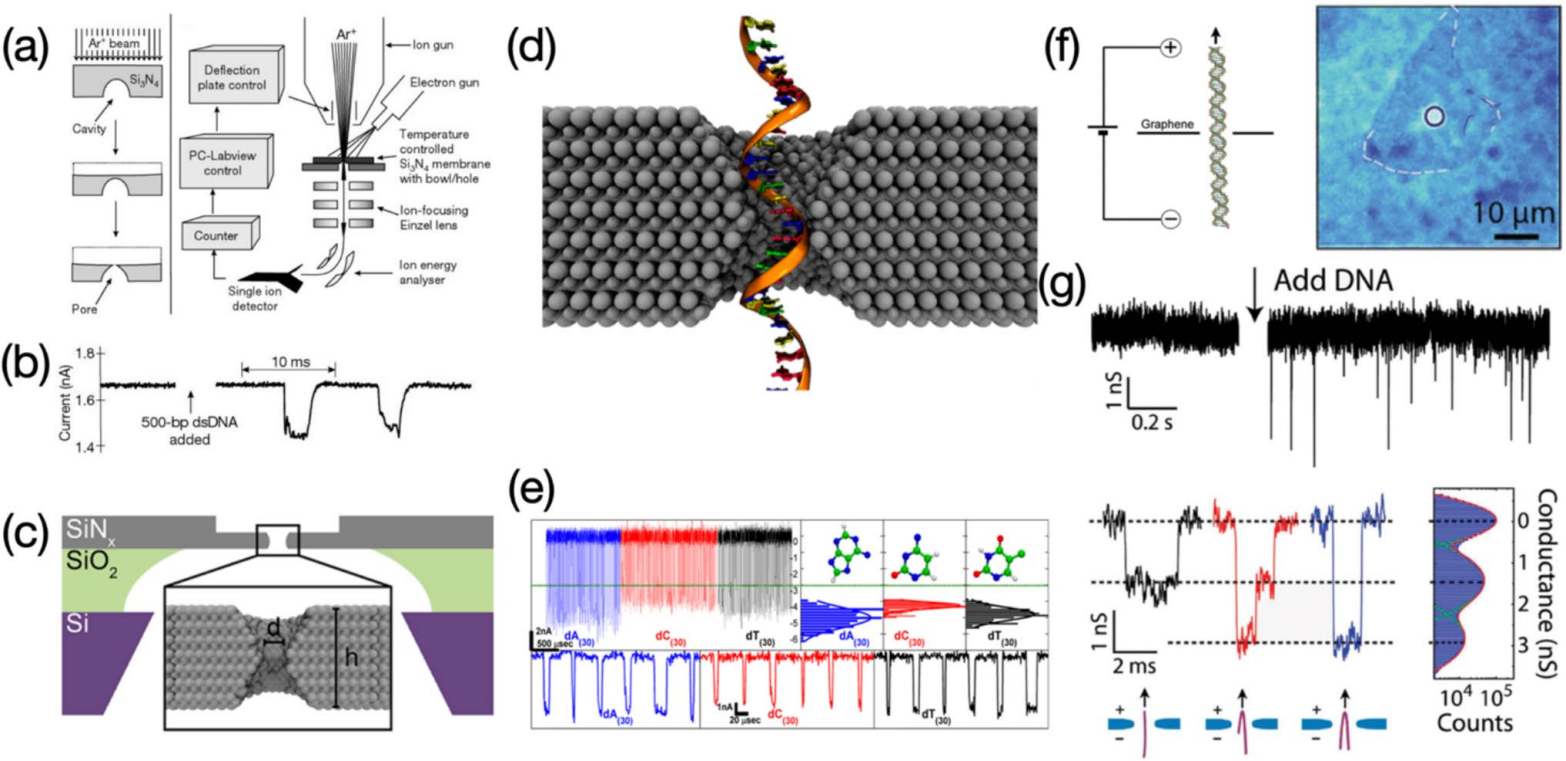
Solid-state nanopores used for DNA sequencing: **a** Schematic illustration of a Si3N4 nanopore via argon ion-beam sculpting. Image reprinted with permission from Li et al. (2001); **b** dsDNA translocation event recorded. Image reprinted with permission from Li et al. (2001); **c** SiN_*x*_ nanopore fabricated on a large aperture-containing Si/SiO_2_ substrate. Image reprinted with permission from Venta et al. (2013); **d** ssDNA threading through the SiN_*x*_ nanopore. Image reprinted with permission from Venta et al. (2013); **e** DNA homopolymers translocate through prepared SiN_*x*_ nanopore and comparison of current levels among dA_(30)_, dC_(30)_, and dT_(30)_. Image reprinted with permission from Venta et al. (2013); **f** Schematic diagram of DNA sequencing on a graphene nanopore. Image at right-hand side reveals a piece of graphene layer transferred onto a silicon nitride chip containing a 5 μm aperture. Image reprinted with permission from Schneider et al. (2010); **g** Translocation signals upon drilled graphene nanopore, along with DNA translocation events with different spatial conformation and corresponding statistical analyses. Image reprinted with permission from Schneider et al. (2010)

DNA sequencing based on solid-state nanopores usually faces the challenge of drilling nanopores with a diameter at round 1 nm. The first solid-state nanopore was successfully produced within a thin layer of Si_3_N_4_ membrane containing a bowl-shaped depression on the other side, via the ion-beam sculpting (Fig. 12a) (Li et al. 2001). The blockage against ion current was verified when dsDNA threaded through the Si_3_N_4_ nanopore with a diameter of 5 nm (Fig. 12b). This work opens a new path for characterization of DNA molecules based on inorganic nanodevices. Nanopores with further reduced diameters (i.e., 0.8 ~ 2 nm) were fabricated by drilling the thin layer of SiN*x* (thickness of 5 ~ 8 nm) coated on the Si/SiO2 chip (Fig. 12c), using as nanosensors for assessing DNA molecules (Fig. 12d). (Venta et al. 2013) The constriction domain of the SiN*x* nanopore featuring a mean diameter of 1.2 nm and a length of 1.7 nm is highly comparable to those in commonly used porin-based nanopores. DNA homopolymers that contain 30 bases, including poly (dA), poly(dC), and poly(dT), were tested using the nanopore, followed by a statistical analysis on numerous threading signals to achieve the base discrimination (Fig. 12e). However, the presented open-pore baseline is thick probably due to intrinsic large background noise of solid-state nanopores. Moreover, only the signal of poly(dA) is differentiable from other homopolymers according to the magnitude of current blockage as there is no apparent difference observed between the signal of poly(dC) and poly(dT). Nanodevice fabrication based on two-dimensional (2D) materials has been becoming a hot topic since Novoselov and Geim first obtained a clean graphene sheet peeled off from a bulky graphite using the ‘scotch tape’ method in 2004 (Novoselov et al. 2004). Garaj et al., then claimed graphene can be applied as a trans-electrode membrane with a thickness below 1 nm (Garaj et al. 2010). By drilling a nanopore (diameter of 22 nm) within a single layer of graphene on a Si/SiO_2_ chip containing a 5 μm aperture, Schneider et al., aimed to provide proof of concept the possibility of DNA sequencing could be realized on nanopores within atomically thick membranes (Fig. 12f) (Schneider et al. 2010). Up to 1,222 events of DNA translocation presented as conductance signals were statistically recorded revealing different behaviors, involving the open-pore (i.e., blank), non-, partial-, and entire- folding (Fig. 12g). Same as the work of Venta et al., there was no effort on the reduction of nanopore noise, resulting in a thick open-pore baseline (Fig. 12g). Merchant et al. reported a graphene single layer supported on a tandem SiN/SiO_2_/Si composite, along with a nanopore drilled in the graphene plane for DNA translocation (Merchant et al. 2010). Diameters of nanopores created were about 5 ~ 10 nm, they were suitable for DNA translocation while maybe not very appropriate for reading DNA sequences. However, these works are still regarded as a pioneering attempt in solid-state nanopore based DNA sequencing. For future development, enzymatic components such as DNA polymerase or helicase can be introduced to overcome the challenge in reading sequences under an extremely high translocation speed.

**Fig. 13:**
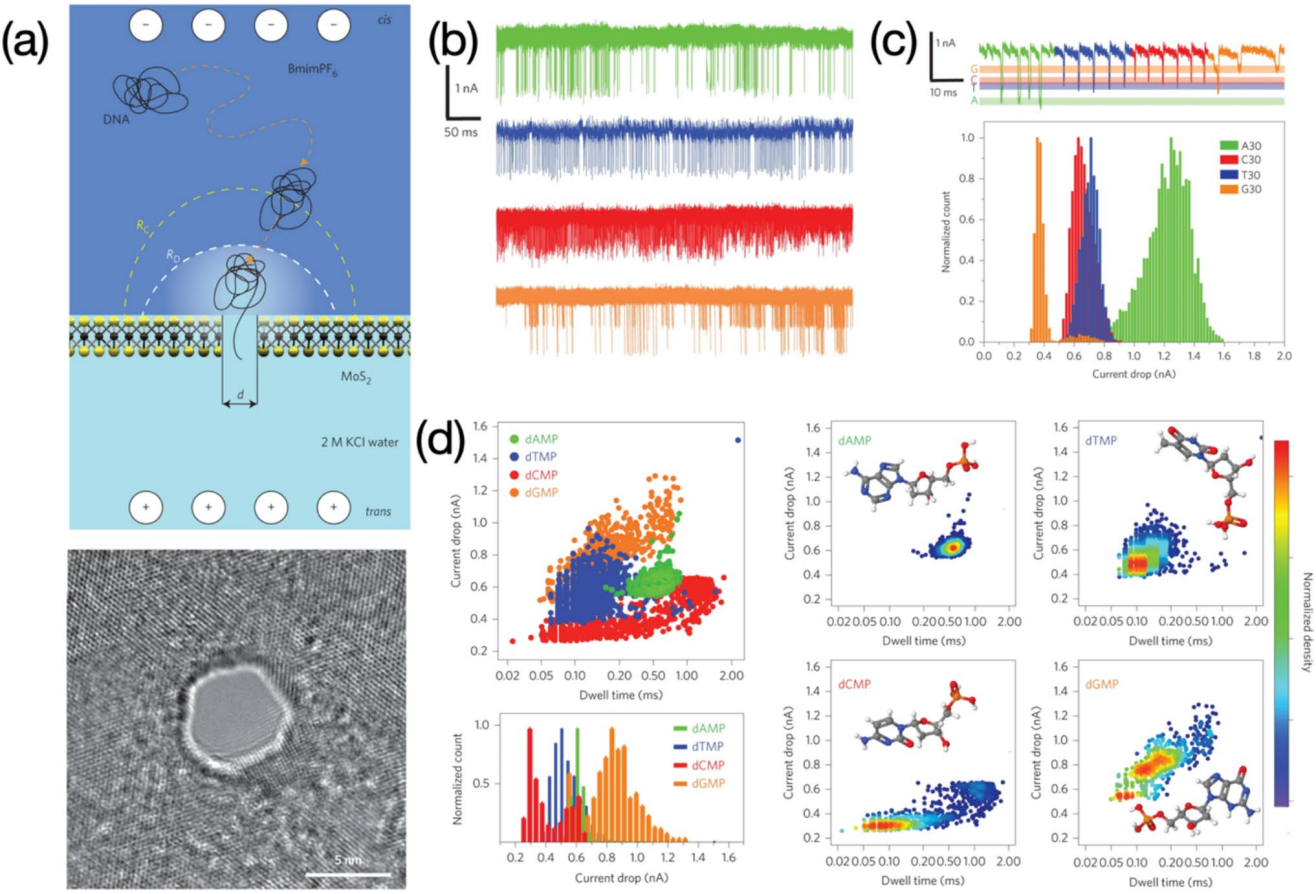
DNA sequencing based on a single layer of MoS_2_: **a** Detection pool separated by a MoS_2_ membrane containing a nanopore, where *cis-* area is loaded with an ionic liquid of BmimPF_6_ and *trans-* area is loaded with KCl aqueous solution, along with a TEM image of 5 nm nanopore created within the monolayer of MoS_2_; **b** Translocation events of DNA homopolymers; **c** Statistical evaluation upon current blockages of A30, T30, C30 and G30; **d** Statistical analysis upon dAMP, dTMP, dCMP, and dGMP translocation events, of which data revealing current drops versus dwell times are presented in both integrated and individual ways

Aleksandra Radenovic et al. reported progress on solid-state nanopore sensing regarding discrimination of four types of nucleotides via a nanopore manufactured in a MoS_2_ membrane (Fig. 13a) (Feng et al. 2015). The detection pool was separated by the MoS_2_ membrane, having the ‘*cis*’ compartment loaded with viscous room-temperature ionic liquid (RTILs) of 1-butyl-3-methylimidazolium hexafluorophosphate (BmimPF_6_), while the ‘*trans*’ compartment loaded with KCl aqueous solution. Electrophoretic and electroosmotic forces offered a synergistic ‘push-like’ effect upon DNA molecules once they were trapped within the capture radius (R_c_) of the MoS_2_ pore (Fig. 13a). The use of BmimPF6 can effectively slow down the DNA translocation since the highly viscous ionic liquids provide DNA strands with intense Stokes dragging force and keep them as an entwined configuration before passing through the nanopore (Feng et al. 2015). Two classes of DNA analytes including DNA homopolymers (30-mers) and deoxynucleotides (monophosphates) were tested using the MoS_2_ nanopore, yielding downward spikes with different amplitudes to identify the four nucleobases (Fig. 13b, d). The dwell time, another significant nature in nanopore sensing, was also assessed using deoxynucleotides tested, from which results yielded (e.g., current drop versus dwell time) were plotted in both merged and separate ways (Fig. 13d). Separated plotting contains points with different densities revealing the reappearing frequency of current blockages related to each deoxynucleotide. This work is a successful example of the use of thin film nanopores to determine biological molecules. However, there are still several issues required to be addressed before reaching the destination of continuous DNA sequencing at high resolution: 1) nanopores with reduced diameters of 1 ~ 1.2 nm are more suitable for high resolution DNA reading, while the size of the nanopore fabricated within the MoS_2_ membrane shown in this work is 5 nm; 2) there is lack of attempts on successive reading of a strand with random base sequence, although the ionic liquid of BmimPF6 is applied to remarkably reduce the translocation speed of DNA at the current work; 3) the current-drop values, either for 30-mer homopolymers (e.g., A30, T30, C30, and G30) or for deoxynucleotides (e.g., dAMP, dTMP, dCMP, and dGMP), are discriminated depending on counting peaks. However, from a view of comparison, signals in relation to these two types of biomolecules are not very consistent, which are hard to realize reasonable identification on current blockages reflecting each base particularly in random base sequencing (Fig. 13c, d).

With the development of computer science, an increasing number of study groups start to use computer-aided simulation or mathematical modeling to current signals and potential interactions yielded when DNA sequences thread through solid-state nanopores, especially for ones created within 2D thin films. Wells et al. theoretically studied ssDNA molecules passing through a graphene nanopore based on all-atom molecular dynamics and atomic-resolution Brownian dynamics (Wells et al. 2012). Likewise, Zhang et al., used molecular dynamic simulations to investigate DNA strands threading through the nanopore created within a monolayer of hexagonal boron nitride (h-BN) for the single-base identification (Zhang and Wang 2016). Moreover, all-atom steered molecular dynamics (SMD) simulations can provide information about effects of nanopore geometries within a graphene film on DNA sequencing. (Zhang et al. 2014). Characterizations on intrinsic properties of a novel layer with atomic thickness such as graphdiyne (GDY) and status of four types of nucleobases threading through its in-plane nanopore can be achieved by the first-principles density functional theory (Kumawat and Pathak 2021). Furthermore, this method can be also applied to evaluate different spatial conformations of adenine (A), cytosine (C), guanine (G), thymine (T) and uracil (U), when they pass through a graphene nanopore, as well as their effects on ssDNA or RNA sequencing (Nelson et al. 2010). Nonetheless, the transformation from computational analyses to practical applications (e.g., high-resolution DNA sequencing) for solid-state nanopores is still the common aspiration of scientific researchers all over the world, although the detection of DNA molecules via theoretical studies have realized in-depth development.

## Conclusions and prospects

As the review presents, functionalized electrode-based sensors and nanopores are two primary nanodevices used for electrochemical assessment of DNA molecules. Functionalized electrode-based sensors are often equipped with functional layers highly effective to adsorption of DNA probe sequences for sensitive detection of target DNA, providing outcomes of electrochemical responses varied with analyte concentrations. Voltammetric (e.g., DPV) and impedimetric (e.g., EIS) measurements are mostly used for trace amount detection of DNA molecules, while other electrochemical techniques like CV, SWV, and LSV are also frequently reported. Detection results reveal both positive and negative correlation between concentrations of target DNA sequences and electrochemical signals owing to use of different sensing mechanisms. Redox couples (e.g., [Fe(CN)_6_]^3−/4−^) against a synergistic resistive effect made of the probe-target steric hinderance and negative charge repulsion usually lead to a typical negative correlation, while electroactive indicators (e.g., MB) intercalating into structural intervals formed after the probe-target hybridization promote the electron transfer, resulting in a positive correlation.

Although functionalized electrode-based sensors in combination with electrochemical techniques often achieve outstanding performances on determination of DNA such as wide dynamic range, ultralow LOD, high selectivity, good reproducibility, long storability, and reliable real sample detection, majority of them merely exhibit a single function of concentration quantification. Multiplexed electrodes that can simultaneously detect two or even more DNA analytes and versatile sensors capable of screening not only DNA strands but also macromolecules (e.g., proteins) are probably in large demand in the future. Also, flexible electrodes based on inexpensive soft substrates and electrodes integrated with novel 2D materials (e.g., MXenes) have potent advantages of low cost, mass production, large surface area and light weight, which are competitive alternations that replace conventional hard electrodes (e.g., GCE) in near someday.

Nanopores are a class of special electrochemical nanodevices that are able to decipher bioinformation relying on nucleobase-dependent current blockages when DNA sequences thread through the nanopore driven by an electrical field force applied. Up to date, high-resolution DNA sequencing is only realized on porin-based nanopores. α-HL and MspA nanopores as well as their mutated variants are mostly used transmembrane channels in comparison to other porins. Continuous reading of a DNA strand with clear “stair-like” current signals can be obtained from these porin-based nanopores coupled with enzymatic translocation speed controllers (e.g., DNA polymerases or helicases). However, in actual studies, double-layered phospholipid membranes are highly influenced by temperature, humidity, and solution osmatic pressure. Extremely thick phospholipid membrane often leads to failure of porin embedding, while ultrathin phospholipid membrane easily causes break-up or multiple porin embedding under a certain voltage. Polymeric membranes recently have been attracting a great amount of attention owing to strengths of good stability, high durability, and membrane-forming efficiency, which may be employed as the next generation membrane for porin-based nanopores.

Several studies have made great effort to recognize DNA homopolymers or deoxynucleotides through solid-state nanopores, other studies also have achieved evaluation on structural variations or motion behaviors of individual nucleobases as they pass through 2D membrane-based nanopores using mathematical modeling and computer-assisted machine learning, there is hitherto no report of a milestone on continuous sequencing of DNA at single-base resolution making use of solid-state nanopores. This is largely attributed to inherent issues upon solid-state nanopores that result in current signals being hard to differentiate and analyze. For example, the challenge of high background noise or low SNR mainly sourced from large total capacitance of entire nanopore-containing chips or in-plane lattice vibration of solid materials appears almost in all types of solid-state nanopores. A probably feasible method worth performing is introducing an extra dielectric layer since successive layers can mimic tandem capacitance connection left a reduced total capacitance. Fragasso et al., offered a nice review on the comparison of current noise in biological and solid-state nanopores (Fragasso et al. 2020). Other strategies for lowering the background noise of nanopores are presented in their work. Moreover, unlike porin-based nanopores that always can couple with DNA-related enzymes to slower the transverse speed of DNA, there is extremely hard to anchor such enzymatic components precisely nearby solid-state nanopores. Nanopores created within functional group-containing materials (e.g., graphene oxide) or metal organic frames (MOFs) may somehow address this issue since functional groups or active metal cores exhibit strong affinity towards enzymatic biomolecules via either covalent or non-covalent adsorption. Furthermore, the dimension of solid-state nanopores obtained from current pore-making techniques are unable to achieve an atomic-grade accuracy like porin-based nanopores due to the sub-nano-level atom arrangement of solid materials. This is currently considered as one of the most difficult technical bottlenecks. Creating a pore-diameter of about or sub 1 nm within a single 2D membrane is possibly accessible, but creating solid-state nanopores with configurational identity at atomic level is extremely hard based on commonly used pore-drilling techniques. This drawback can be overcome through a computational algorithm or an alignment with consensus sequences to rectify current-blocking levels of nucleobases from batch-to-batch measurements. However, the work of signal collection and data analysis would be substantially increased. Following the trend of nanopore studies, porin-based nanopores still dominate the currently laboratory and commercialized DNA sequencing technologies. Besides, there are increasing number of studies on peptide or organic small molecules sequencing based on porin-based nanopores. Unlike negatively charged DNA molecules, electron carriage of tested peptides becomes more complicated. Some scientific groups made great achievements on designing different mutation sites near the constriction area of protein nanopores aiming to increase the signal resolution by introducing the electroosmotic flow (EOF) (Yu et al. 2023; Martin-Baniandres et al. 2023; Sauciuc et al. 2024; Liu et al. 2022). Porin-based nanopores in the future would be with more various modifications catering for different purposes, while solid-state nanopores are still on the way to address technical bottlenecks before they are deemed as a counterpart of porin-based nanopores someday.

In a view of comparison, functionalized electrode-based sensors are more suitable for quantitative analysis of DNA than nanopores. While nanopores exhibit more strengths than functionalized electrode-based sensors when they are used to decipher information of bases in a DNA strand. Functionalized electrode-based sensors have advantages of cost effectiveness, easy to use, less-time responses, setup miniaturization, and onsite application. However, most of them are only with a single function of target quantification reflecting concentration results after recognized with target-specific DNA probes. Nanopores instead can discriminate multiple targets via base-dependent current levels. However, the target quantification based on nanopore technologies is somehow less effective than functionalized electrode-based sensors since “spike-like” signals obtained from the nonenzymatic pore-threading are unable to accurately reveal the amount of DNA strands passing through a nanopore within a fixed duration. Moreover, the constriction area of porin-based nanopores only allow biomolecules with cross-sectional dimensions about or less than 1 nm to pass through, which may be more friendly to “chain-like” molecules without secondary or even more complicated structures.

Functionalized electrode-based sensors integrated with microfluidic systems achieving multiplex target monitoring may be in high demand in the future (Ghorbanpoor et al. 2022). Equipped with increasingly higher sensitivity, this type of sensor may be very valuable in early diagnosis of nucleic acid-related diseases and determination of toxins in water or soil specimens from natural environments. To further reduce the fabrication cost, soft substrates may be preferred in future fabrication of functionalized electrode-based sensors though a massively produceable printing process. Moreover, majority of soft substrates own properties of light weight and biocompatibility, which make them highly prone to be integrated into hand-carry devices or be turned into wearable smart sensors. Future nanopore technologies may advance DNA analysis in clinical or environmental applications by increasing the accuracy of DNA base reading. DNA sequencing results yielded from currently used porin-based nanopores are always limited by the configuration of porins, producing current signals are co-influenced by adjacent 3 ~ 5 bases. Lipid bilayers can be formed on a cheap aperture-containing microchips (e.g., printed circuit board), providing appropriately membranous environment for embedment of protein nanopores. These microchips can be subsequently integrated into a portable sequencing device for real-time DNA recording at various circumstances. Nanopore arrays enable dozens to hundreds of nanopores to be massively created within a pre-designed area for high-throughput DNA sequencing, thus reducing the cost of sample preparation and testing. According to recent advances and future perspectives, next generations of either functionalized electrode-based sensors or nanopores require to be developed into more effective, cheaper and smarter forms to meet growingly higher demands of human beings.

## Data Availability

No datasets were generated or analysed during the current study.
